# Developing Pericarp of Maize: A Model to Study Arabinoxylan Synthesis and Feruloylation

**DOI:** 10.3389/fpls.2016.01476

**Published:** 2016-09-30

**Authors:** Anne-Laure Chateigner-Boutin, José J. Ordaz-Ortiz, Camille Alvarado, Brigitte Bouchet, Sylvie Durand, Yves Verhertbruggen, Yves Barrière, Luc Saulnier

**Affiliations:** ^1^BIA, INRANantes, France; ^2^National Laboratory of Genomics for Biodiversity (Langebio-CINVESTAV), Mass Spectrometry and Metabolomics LabIrapuato, Mexico; ^3^UGAPF, INRALusignan, France

**Keywords:** grass cell wall, maize grain, xylan, ferulic acid, *p*-coumaric acid, phenylpropanoid pathway, BAHD feruloyltransferase, peroxidase

## Abstract

Cell walls are comprised of networks of entangled polymers that differ considerably between species, tissues and developmental stages. The cell walls of grasses, a family that encompasses major crops, contain specific polysaccharide structures such as xylans substituted with feruloylated arabinose residues. Ferulic acid is involved in the grass cell wall assembly by mediating linkages between xylan chains and between xylans and lignins. Ferulic acid contributes to the physical properties of cell walls, it is a hindrance to cell wall degradability (thus biomass conversion and silage digestibility) and may contribute to pest resistance. Many steps leading to the formation of grass xylans and their cross-linkages remain elusive. One explanation might originate from the fact that many studies were performed on lignified stem tissues. Pathways leading to lignins and feruloylated xylans share several steps, and lignin may impede the release and thus the quantification of ferulic acid. To overcome these difficulties, we used the pericarp of the maize B73 line as a model to study feruloylated xylan synthesis and crosslinking. Using Fourier-transform infra-red spectroscopy and biochemical analyses, we show that this tissue has a low lignin content and is composed of approximately 50% heteroxylans and approximately 5% ferulic acid. Our study shows that, to date, maize pericarp contains the highest level of ferulic acid reported in plant tissue. The detection of feruloylated xylans with a polyclonal antibody shows that the occurrence of these polysaccharides is developmentally regulated in maize grain. We used the genomic tools publicly available for the B73 line to study the expression of genes within families involved or suggested to be involved in the phenylpropanoid pathway, xylan formation, feruloylation and their oxidative crosslinking. Our analysis supports the hypothesis that the feruloylated moiety of xylans originated from feruloylCoA and is transferred by a member of the BAHD acyltransferase family. We propose candidate genes for functional characterization that could subsequently be targeted for grass crop breeding.

## Introduction

There is a need to better understand grass cell walls as they impact significantly upon mankind daily life and are of economic importance. Cell walls are widely used in a multitude of food, feed, and non-food industries. For example, they can serve as a raw diet for herbivore cattle or as a raw material for the production of biofuel or “green-based” products. Grass cell walls are extracellular matrices mostly comprised of polymers that cross-link together to form a complex network and are implicated in most of the physiological processes of plants, including, for example, plant development and plant defense. The major hemicellulosic polysaccharides found in grass cell walls are xylans. Distinct polysaccharides are encompassed in the xylan family and share, as a feature, a backbone comprised of a linear chain of β-1,4-linked d-xylopyranosyl residues. There are large variations in the degree and nature of the substitutions present on the xylan backbone, which depend on plant species, cell type, and developmental stage. Side chains of xylans in grasses are commonly composed of acetic acid and the following sugars: α-d-glucuronic acid, 4-*O*-methyl glucuronic acid, galactopyranosyl, xylopyranosyl and arabinofuranosyl residues (Ebringerova et al., 2005; Peña et al., 2016). It is important to note that xylans from grasses differ from those of eudicots (Peña et al., 2016). In grasses, xylans have a higher content of arabinose substitution than in eudicots. Moreover, unlike eudicots, grass xylans possess hydroxycinnamic acids, mainly ferulic acid and *p*-coumaric acid, which are linked to either single arabinose residues or to arabinose residues present in oligosaccharide side chains (Saulnier et al., 1995a; Schendel et al., 2016). Another feature of xylans present in grasses is the absence of the tetra-saccharide sequence found at the reducing end of xylan in eudicots and gymnosperms. By contrast, xylose residues present at the reducing end of grass xylans have been reported to be substituted at *O*-2 by 4-*O*-methyl glucuronic acid or at *O*-3 by arabinofuranose residues (Ratnayake et al., 2014; Peña et al., 2016).

In the cell walls of grasses, ferulic acid is attached to the arabinose residues of xylans via an ester linkage. Under oxidative conditions, chains of arabinoxylan can cross-link through a ferulate bridge that forms either ferulate dehydrodimers, trimers or tetramers (Hatfield et al., 1999; Saulnier and Thibault, 1999; Rouau et al., 2003; Bunzel et al., 2004). Ferulic acids can also attach to lignins via ether bonds, and it has been shown that lignins and arabinoxylans can bind covalently to each other through the ferulate molecule (Jacquet et al., 1995). Ferulate bridges are thought to be important structural components of grass cell walls. It has been suggested that they contribute to grass wall assembly, promote tissue cohesion, restrict cell expansion, contribute to the mechanical properties of mature tissues, and participate in plant protection against pathogens (for review, see Buanafina, 2009). The presence of ferulate impedes the degradation of plant tissues and therefore has a negative impact on various industrial processes as well as silage digestibility (Grabber et al., 1998; Chundawat et al., 2011; Jung et al., 2011). *p*-Coumaric acid is another hydroxycinnamic acid that decorates grass arabinoxylans and lignins. Although it can acylate both polymers, its ability to cross-link these polymers through oxidative conditions *in planta* has not been clearly demonstrated (Ralph et al., 1994; Sibout et al., 2016). A minor amount of *p*-coumaric acid can undergo photocatalyzed cyclodimerization to form truxillic and truxinic acids (Hartley et al., 1990), and their impact on cell wall properties is thought to be limited.

Arabinoxylan biosynthesis requires numerous enzymes to build the xylan backbone and add the different substitutions. Many genes involved in the biosynthesis of xylans have recently been identified using the genomic tools available for *Arabidopsis thaliana* (for review, see Rennie and Scheller, 2014). By contrast, only a few genes have been identified in grass species. In wheat and rice, several genes involved in the backbone synthesis have been discovered (Chen et al., 2013; Lovegrove et al., 2013; Jiang et al., 2016; Zeng et al., 2016). To date, however, only two glycosyltransferases responsible for the substitution of the xylan backbone by arabinose and xylose, respectively, have been reported (Anders et al., 2012; Chiniquy et al., 2012). A “blastp” strategy, similar to that carried out by Courtial et al. (2013), could contribute to the discovery of enzymes involved in the incorporation of other substitutions of the xylan backbone in maize. However, such an approach, based on sequence similarity, is restricted to the data available for other species and consequently does not allow us to identify gene/protein candidates for the several xylan substitutions that have no assigned proteins.

Similar to the lignin monomers, the hydroxycinnamic acids ferulic acid and *p*-coumaric acid, which are ester-linked to arabinoxylans, are derived from the phenylpropanoid pathway. The main steps and enzymes required for the production of lignin monomers and *p*-coumaric acid have been largely deciphered. However, new steps have recently been discovered and have modified our understanding of lignin biosynthesis (see, for example, Vanholme et al., 2013; Wang et al., 2013). Several steps leading to the formation of feruloylated arabinoxylans are still under debate. Ferulic acid, feruloylCoA and feruloylglucose have all been suggested as feruloyl donors in the formation of feruloylated arabinoxylans. Likewise, it is unknown (i) if the acceptor of this transfer is arabinoxylan or UDP-arabinose, (ii) if the transfer occurs in the cytosol, the Golgi or the cell walls, and (iii) which enzymes are involved in the binding of ferulic acid to arabinoxylans (Saulnier et al., 2012; Molinari et al., 2013). One current model proposes that the feruloylCoA produced by the phenylpropanoid pathway is the active form of ferulic acid that is transferred to arabinoxylans or UDP-arabinose by members of the BAHD superfamily of acyltransferases (Mitchell et al., 2007; Molinari et al., 2013). The BAHD superfamily, also referred as Pfam family PF02458 (D'Auria, 2006), has been divided into eight clades (Tuominen et al., 2011), with some clades over-represented in rice compared with Arabidopsis (Mitchell et al., 2007). Since feruloylated arabinoxylans are present in the cell walls of rice but are absent in Arabidopsis, these clades were proposed as candidates for feruloyltransferases acting on arabinoxylans. Feruloylated arabinoxylans are transported to cell walls by exocytosis. Oxidative coupling of ferulic acid occurs intracellularly and in the cell walls to produce dimers and oligomers, and it has been suggested that class III peroxidase is involved in the process (Fry et al., 2000; Burr and Fry, 2009).

Based on previous studies, we postulated that maize pericarp is a relevant model to study ferulate and arabinoxylan biosynthesis, ferulate transfer to arabinoxylans and ferulate dimerization. Our studies conducted on maize bran, which is mostly constituted by the pericarp and is a by-product of the maize processing industries, have shown that arabinoxylans represent approximately 50% of the bran weight (Saulnier et al., 1995b). In their study on ferulate ester distribution, Harris and Trethewey (2010) reported that maize grain was the most abundant source of ferulate ester (20 mg.g^−1^ cell walls). We have shown that, in bran, the amount of ferulic acid ester linked to cell wall arabinoxylans can reach up to 35 mg. g^−1^ of dry matter (3.5%) (Chanliaud et al., 1995; Saulnier et al., 1995b). In maize bran, the structure of xylans has been extensively documented (Chanliaud et al., 1995; Saulnier et al., 1995b; Allerdings et al., 2006; Agger et al., 2010; Schendel et al., 2016), and its lignin content has been reported to be low (minimum 1%) (Chanliaud et al., 1995; Saulnier et al., 1995b; Lapierre et al., 2001). Taken together, these results facilitate the study of feruloylated arabinoxylans. Moreover, with the availability of its sequenced genome, publicly available gene expression data collected in a dedicated database (the Maize Genetics and Genomics database; MaizeGDB), mutant collections and protocols to generate genetically modified maize, the B73 maize line is a good model for functional genomics.

To study grass cell walls and especially the biosynthesis of feruloylated arabinoxylans, we selected the maize grain pericarp as a model. In this report, the feruloylated arabinoxylan content of grain tissues (pericarp and endosperm) from the maize B73 line was measured and compared with those of 18 other maize lines. We determined the stages of development where the deposition of feruloylated arabinoxylans occurs in B73 grains, and we have used published and publicly available data to monitor the expression of genes associated with feruloylated arabinoxylan and lignin (Sekhon et al., 2011; Courtial et al., 2013; Barrière et al., 2016). Our study of maize pericarp highlights the expression of cell wall genes involved in the synthesis of feruloylated arabinoxylans, and as a consequence, we propose several robust candidate genes involved in ferulate and arabinoxylan synthesis and in the feruloylation and the cross-linking of xylans.

## Materials and methods

### Maize lines

The lines investigated in this study were chosen as representative of maize genetic diversity and earliness and are related to the Iowa Stiff Stalk Synthetic group (B37, B73, A632), Lancaster group (Mo17, FR600, C103), Reid Yellow Dent group (WF9, W64A, Oh03, A654), Minnesota13 group (W117), Wisconsin25 and Golden Glow group (W401, W182E, F113), flint and/or dent Canadian group (CM7, F252), European flint (F2), and two miscellaneous lines (CO255, ILO863). Maize lines were grown in Aubiat (France) in 2004, and grains were kindly provided by Limagrain. B73 seeds were also obtained from INRA Saint Martin de Hinx (France). The line B73 was used in the model study.

### Growth and harvest conditions

Maize B73 plants were grown in 2014 at INRA Angers (France) on soil in a glass-house under a 16-h light/8-h dark cycle at 19°C and 17°C, respectively. Maize ears were tagged at silk appearance. The plants were hand-shaken during silk elongation to favor pollination, and grain development was assessed as days after pollination (DAP). Grains were collected from the central part of the ears. To compare our samples with the samples analyzed by Sekhon et al. (2011), depicted in the Maize eFP Browser of the MaizeGDB, we used the same system of annotation to classify the developmental stages of the grains. The reproductive R stage annotation of the Iowa State University method (Hanway and Ritchie, 1984) used in this study is as follows: R0: silking; R1: silked; pre-blister: brown silks, no fluid in the grain; R2: blister, the grain contains fluid but no starch; R3: milky endosperm with starch; R4: doughy endosperm; early dent: the grains begin to dent; R5: most grains are dented; Nearly mature: grains are drying, shiny but not hard; R6: mature. Coloration with an iodine potassium iodide (I2-KI) solution (0.25% iodine dissolved in a 0.4% aqueous solution of potassium iodide) applied to grain sections was used to observe the presence of starch and to distinguish the R2 and R3 stages. Longitudinal sections (200 μm) of grains were obtained using a vibratome (HM 650V, MICROM) and were observed using a Multizoom macroscope (AZ100M, NIKON) under bright-field conditions or epifluorescence irradiation using a band-pass filter (325–375 nm) as an excitation filter and fluorescence detection above 420 nm.

To collect primary roots, maize B73 plants were grown in the dark at 23°C for 7 days.

### Microscopy

**Figure 1:**
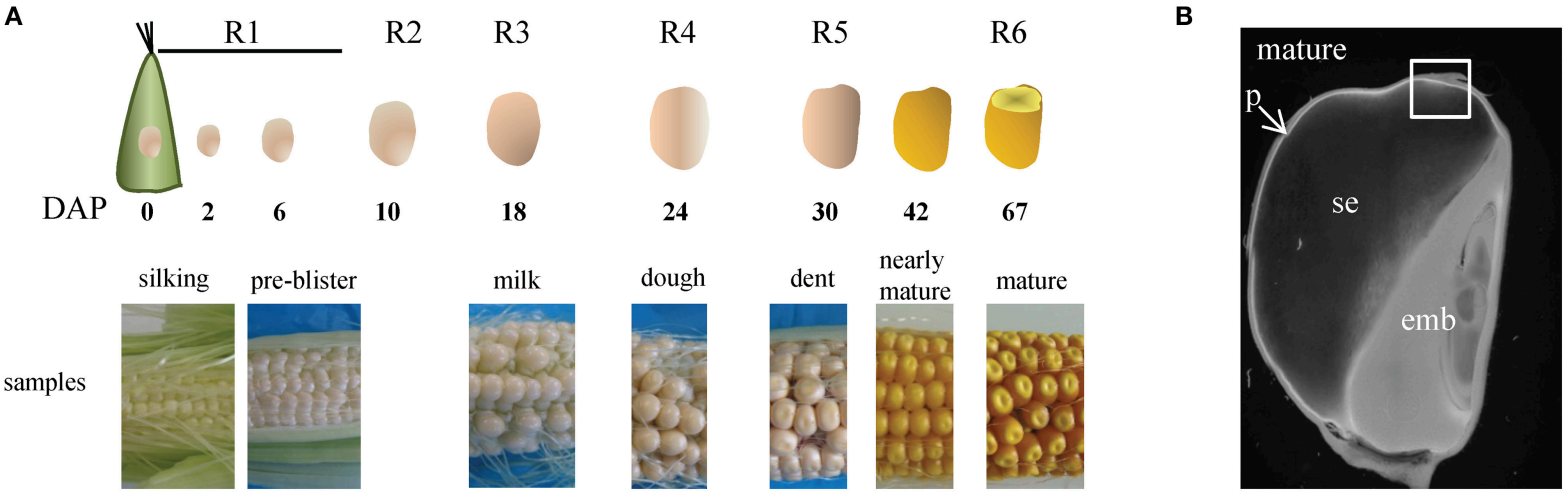
**Maize B73 grain during development and at maturity. (A)** Stages of grain development from ear silking to mature grain. The stages are defined as Days After Pollination (DAP) and according to the R stage annotation of the Iowa State University method (Hanway and Ritchie, 1984). **(B)** Longitudinal section of a mature maize grain observed with a fluorescence macroscope. Square: sample collected for toluidine blue staining and immunolabeling. p, pericarp; se, starchy endosperm; emb, embryo.

For immunolabeling and toluidine blue staining of grains, pieces were cut from the upper part of the caryopsis in the vicinity of the silk attachment point (Figure 1) within 2 h after harvest. The samples were immediately incubated in a fixative solution (0.5% glutaraldehyde, 3% paraformaldehyde) overnight and subsequently embedded in London Resin White acrylic as described in Chateigner-Boutin et al. (2014), which allows samples to be stored for several months. Once fixed samples were obtained for all developmental stages, they were cut into semi-thin sections (1 μm, ultracut UC7, LEICA). Sections were stained with toluidine blue O (1% in 2.5% Na_2_CO_3_) for 5 min as performed in Chateigner-Boutin et al. (2014) or labeled with a polyclonal antibody (Anti-5OFerAra) that targets a 5-*O*-feruloylated arabinose epitope (Philippe et al., 2007). Immunolabeling was carried out as described in Chateigner-Boutin et al. (2014). The sections were incubated with Anti-5OFerAra diluted 1:1500 in a phosphate buffered saline (PBS)-Tween buffer (0.1 M PBS supplemented with 1% BSA and Tween 20 0.05%) for 1 h at room temperature. Controls were performed under the same conditions, but pre-immune sera were used instead of Anti-5OFerAra (Supplementary Figure 1). The sections were washed extensively in the PBS-Tween buffer and then incubated in darkness for 1 h at room temperature with goat anti-rabbit IgG conjugated with Alexa Fluor 546 diluted 1:100 (v/v) in PBS-Tween buffer. Grain samples representative of each stage of development were simultaneously labeled, and the sections were observed under a fluorescence microscope Leica (DMRD LEICA) using the same settings. A band-pass filter (515–560 nm) was used as an excitation filter, and fluorescence was detected above 590 nm.

Root segments from 7-day-old seedlings were harvested in two distinct regions along the primary roots (elongation and post-elongation zones). The elongation zone was the region sectioned from 0.2 to 0.8 cm distal to the apex, and the post-elongation zone was 5.0–5.6 cm distal from the root apex. For each subset, five excised root regions were immediately fixed overnight in 4% paraformaldehyde in PBS (pH 6.9) at 4°C. The roots were embedded in 7% agarose (only to facilitate sectioning). Transverse sections of 60 μm thickness were produced using a vibratome (HM 650V, MICROM). Immunolabeling was carried out as described for grain sections except that the dilution of the Anti-5OFerAra was 1:500, and the secondary antibody was conjugated with Alexa Fluor 488. Root sections were observed using a macroscope (AZ100M, NIKON) under bright-field conditions and under epifluorescence irradiation (a band-pass filter of 461–489 nm was used as an excitation filter, and fluorescence was detected above 515 nm). The images were processed using Adobe Photoshop CS6 to show representative images of the immunolocalization observed.

### Biochemical analyses

#### Sample preparation

Maize grains were soaked for 24 h in a steeping solution containing 0.1% SO_2_ and 0.5% lactic acid to facilitate pericarp and endosperm separation. In contrast to the method described in Singh and Eckhoff (1995), the incubation was performed at room temperature. The grain pericarp and endosperm were separated with tweezers and ground using a Freezer/Mill 6700 (SPEX). Alcohol insoluble residues (AIRs) were obtained from 1.5 g of endosperm powder and 0.2 g of pericarp powder by carrying out the following steps. The ground material was mixed with 80% ethanol and heated for 10 min at 100°C prior to centrifugation. The pellet was recovered and rinsed twice with 80% ethanol, once with 95% ethanol and then dried for 24 h at 40°C.

#### Neutral sugar analyses

Following the method of Englyst and Cummings (1988), the AIR samples obtained from the endosperm and pericarp were hydrolyzed with 1 M H_2_SO_4_ for 2 h at 100°C, and their neutral sugars were derivatized into their alditol acetate form to measure their content by gas chromatography. To fully release glucose from cellulose, a prehydrolysis step was carried out with 72% H_2_SO_4_ for 30 min at 25°C prior to the hydrolysis step. The analyses were performed in duplicate and are presented as mean values. The standard error was less than 5%.

#### Ester-linked hydroxycinnamates

Ester-linked phenolic acids were saponified under oxygen-free Ar in 2M sodium hydroxide for 45 min at 35°C. Under these conditions, ferulic acid ether-linked to lignins is not released. An internal standard (2,3,5 trimethoxy-(E)-cinnamic acid (TMCA), T-4002, Sigma Chemical Co., St. Louis, USA) was added before adjusting the pH to 2. Phenolic acids were extracted with diethylether and quantified by RP-HPLC as described by Antoine et al. (2003). The response factors of the following forms of ferulic acid dehydrodimers (diFA) (8-O-4′, 8-5-non-cyclic, 8-5′-benzofuran, 5-5′) and ferulic acid dehydrotrimer (TriFA) (Rouau et al., 2003) relative to the internal standard were determined at 320 nm by comparison with purified samples. The ferulic acid monomer (FAm) content was calculated from the amount of cis- and trans-ferulic acid. The 8-5′ dehydrodimer was calculated as the sum of 8-5′-non-cyclic and 8-5′-benzofuran, and the total amount of ferulic acid dehydrodimers was calculated as the sum of the 8-O-4′, 8-5′, and 5-5′ forms. The analyses were performed in duplicate, and the standard error of the mean was less than 10%.

#### Fourier transform-infraRed (FT-IR) spectroscopy

FT-IR spectra were obtained from AIRs of the maize pericarp and reference samples of maize F2 internodes (F2 line, collected at the silage stage) (Chazal et al., 2014), straw of wheat (Champlein cultivar) and *Brachypodium distachyon pCA monolignol transferase1 (Bdpmt1)* mutant (Petrik et al., 2014).

FT-IR spectra were recorded from KBr pellets made from 2 mg of samples mixed with 120 mg of KBr. The spectra were collected in transmission mode between 4000 and 700 cm^−1^ at 2 cm^−1^ intervals (Thermo Nicolet IS50 spectrometer). The IR spectra resulted from the co-addition of 200 interferograms. All IR spectra in the 2000–700 cm^−1^ region were baseline-corrected and unit vector normalized using OPUS software (version 7). Second-derivative spectral data (Norris Gap, gap size: 9) were processed to enhance spectral differences in the 875–750 cm^−1^ region (Unscrambler 10.1 software, CAMO, Oslo Norway). The second-derivative spectral data were multiplied by −1 and were unit vector normalized. Principal component analyses were applied to the second derivative spectra.

FT-IR band assignments of lignin samples, hydroxycinnamic acids and cell wall polysaccharides were adapted from the literature (Robert et al., 2005; Sebastian et al., 2009; Chazal et al., 2014).

#### Transcriptome analysis

The expression data generated by Sekhon et al. (2011) for 60 maize tissues of the B73 line are publicly available via the MaizeGDB website (www.maizegdb.org) and were used in this study to investigate the expression of genes related to arabinoxylans (AX) and lignins. Robust multiarray average (RMA) normalized data were collected for the 1st internode of the maize stem at stage V7 (7 leaves with visible leaf collars) and the dissected pericarp and endosperm at the R3 stage (18 DAP/R3), together with the maximum absolute signal value. The expression data are expressed as a % of the expression potential, as in Francoz et al. (2015), with the expression potential defined as the maximum absolute signal value in the maize RMA normalized data. The % of the expression potential was color-coded to facilitate visualization of expression differences. As in Sekhon et al. (2011), genes with expression data (RMA normalized value) <200 were considered as not being expressed in the corresponding tissue. Gene expression and co-expressed genes were checked using the same dataset analyzed by the Plant Expression Database PlexdB (www.plexdb.org) as expression profile neighbors using the data for the 60 maize tissues.

The genes investigated here correspond to an updated list of maize genes established by Courtial et al. (2013) and Barrière et al. (2016) with a focus on genes potentially encoding enzymes involved in the synthesis and radical coupling of feruloylated arabinoxylans and monolignols. Maize orthologs to genes whose functions are known in other species were identified by a sequence similarity search based on protein sequences using the BlastP tool of the MaizeGDB with e-value lower than e-75 (Courtial et al., 2013). The list of maize genes for class III peroxidases was obtained from Wang et al. (2015). The list of rice BAHD genes within clades A and B was found in Molinari et al. (2013); their sequences were collected from the Rice Genome Annotation project database (http://rice.plantbiology.msu.edu/). Maize and Brachypodium orthologous sequences of rice BAHD genes were obtained from the Gramene database (http://www.gramene.org/).

## Results and discussion

### Ferulate ester content varies among maize lines and is high in B73 maize pericarp

The pericarp and endosperm from the 19 maize lines were hand-dissected. Their arabinoxylan and ester-linked hydroxycinnamate contents are shown in Table 1 (pericarp) and Table 2 (endosperm).

**Table 1:** **Individual neutral sugars and hydroxycinnamate contents in the pericarp of 19 maize lines**.

**Maize lines**	**Ara**	**Xyl**	**Man**	**Gal**	**Glu**	**pCA**	**FA_m_**	**8-5′**	**8-O-4′**	**5-5′**	**TriFA**	**FA_m_/Ara**	**diFA/Ara**	**FA_m_/di**	**FA/*p*CA**
	**g/100 g of tissue**					**mg/100 g of tissue**						**Molar ratio**			
A632	18.5	31.0	0.3	3.8	17.8	243.8	3864.2	363.6	265.0	202.4	150.9	0.14	0.02	8.3	16.8
A654	17.8	32.3	0.9	4.2	20.1	143.4	3271.9	379.0	266.4	190.4	155.9	0.12	0.02	6.9	25.2
B37	16.0	29.5	0.3	5.8	22.4	200.0	3578.6	335.5	226.5	148.3	92.5	0.15	0.02	9.2	18.5
B73	16.0	30.9	0.4	7.0	21.2	62.6	4011.9	416.8	236.2	215.8	75.2	0.17	0.02	8.7	67.0
C103	18.2	30.8	0.7	4.6	21.6	202.8	3000.6	385.5	341.9	245.4	258.7	0.11	0.02	5.2	17.7
CM7	16.5	27.2	0.7	4.9	23.5	263.1	1935.2	432.1	342.8	205.0	260.1	0.08	0.02	3.3	10.2
CO255	19.5	29.6	0.8	4.8	17.9	51.4	4992.3	275.5	208.3	159.5	106.7	0.17	0.01	13.9	94.4
F113	17.6	28.6	0.8	5.3	20.5	187.2	1888.6	486.9	363.5	207.4	274.2	0.07	0.02	3.0	14.6
F2	20.1	30.4	0.4	4.2	17.9	287.7	3881.0	521.4	386.5	254.8	236.1	0.13	0.02	5.8	15.5
F252	15.9	26.2	0.3	4.3	18.6	96.1	4178.3	450.8	361.6	263.8	229.8	0.18	0.03	6.8	48.3
FR600	18.5	29.1	0.5	5.4	16.9	191.9	3181.9	552.4	330.8	240.9	260.3	0.12	0.02	4.9	20.2
ILO863	16.0	26.7	0.6	4.6	15.8	465.3	2578.2	501.7	415.9	216.0	264.7	0.11	0.03	3.9	7.2
Mo17	14.9	27.1	0.4	3.6	18.8	358.8	3437.5	418.6	305.3	222.1	193.8	0.16	0.02	6.4	10.8
Oh03	18.9	28.4	0.4	5.1	20.2	326.6	3798.5	298.2	218.6	174.7	57.9	0.14	0.01	10.3	11.8
W117	14.1	26.5	0.3	4.8	20.8	278.0	2916.9	330.0	239.0	179.7	134.0	0.14	0.02	6.9	11.6
W182E	17.1	27.6	0.7	5.5	22.6	298.1	3710.4	354.2	256.8	191.8	139.5	0.15	0.02	8.2	13.2
W401	18.6	32.8	0.8	5.0	20.3	177.3	3647.0	409.5	293.4	250.2	186.5	0.13	0.02	6.7	22.8
W64A	17.2	29.1	0.6	6.1	15.2	317.8	4703.6	506.9	366.1	254.4	194.1	0.19	0.03	7.4	16.0
WF9	16.3	30.1	0.3	5.6	17.8	374.9	2422.6	408.6	289.6	209.5	223.9	0.10	0.02	4.6	8.0
Mean	17.3	29.1	0.5	5.0	19.5	238.3	3421.0	411.9	300.8	212.2	183.9	0.14	0.021	6.9	23.7
SD	1.59	1.95	0.21	0.83	2.32	108.8	834.0	77.5	62.5	33.5	69.5	0.03	0.004	2.6	22.5
CV%	9.2	6.7	38.0	16.6	11.9	45.7	24.4	18.8	20.8	15.8	37.8	23.4	20.4	38.2	94.8

*pCA, p-coumaric acid; FA_m_, ferulic acid monomer; diFA, ferulic acid dehydrodimers (8-5′, 8-O-4′, 5-5′); TriFA, ferulic acid dehydrotrimer; FA, total ferulic acid. The B73 line was used in the model study and is highlighted in the table*.

**Table 2:** **Individual neutral sugars and hydroxycinnamate contents in the endosperm of 19 maize lines**.

**Maize lines**	**Ara**	**Xyl**	**Man**	**Gal**	**Glu**	**pCA**	**FA_m_**	**8-5′**	**8-O-4′**	**5-5′**	**TriFA**	**FA_m_/Ara**	**diFA/Ara**	**FA_m_/di**	**FA/*p*CA**
	**g/100 g of tissue**					**mg/100 g of tissue**						**Molar ratio**			
A632	0.67	0.48	0.13	0.11	90.8	0.51	39.8	1.81	1.20	0.71	–	0.041	0.002	21.2	72.0
A654	0.49	0.41	0.14	0.05	78.7	0.27	57.2	3.64	2.20	1.28	–	0.079	0.005	16.0	202.2
B37	0.45	0.34	0.10	0.04	81.2	0.51	29.8	2.22	1.24	0.70	–	0.045	0.003	14.2	56.0
B73	0.68	0.55	0.15	0.11	87.8	0.81	37.5	5.54	2.85	1.72	–	0.037	0.005	7.4	49.7
C103	0.66	0.45	0.11	0.09	77.1	0.24	31.2	2.62	1.64	0.92	–	0.032	0.003	12.0	128.7
CM7	0.59	0.46	0.11	0.10	82.8	0.59	41.0	2.46	1.72	1.01	–	0.047	0.003	15.7	66.6
CO255	0.65	0.54	0.18	0.07	94.9	0.48	74.5	2.00	1.26	0.74	–	0.078	0.002	37.0	139.4
F113	0.84	0.67	0.16	0.12	98.9	0.43	39.5	2.37	1.51	0.80	–	0.032	0.002	16.8	87.4
F2	0.54	0.41	0.14	0.05	79.7	0.66	31.6	3.36	1.95	1.02	–	0.040	0.004	9.9	48.6
F252	0.48	0.41	0.12	0.07	90.4	0.32	42.5	1.71	1.22	0.64	–	0.060	0.003	23.7	121.3
FR600	0.42	0.35	0.13	0.04	97.5	0.26	20.3	1.48	1.00	0.47	–	0.033	0.002	13.7	76.1
ILO863	0.45	0.37	0.10	0.06	89.9	0.59	31.6	2.30	1.22	0.70	–	0.048	0.003	14.9	51.1
Mo17	0.60	0.48	0.11	0.07	76.5	0.39	34.1	3.55	1.92	1.17	–	0.039	0.004	10.2	88.7
Oh03	0.55	0.46	0.15	0.10	91.9	0.51	54.6	2.87	1.77	1.04	–	0.067	0.004	19.1	100.1
W117	0.68	0.50	0.13	0.10	80.9	0.65	49.2	4.78	2.65	1.60	–	0.049	0.005	10.8	75.3
W182E	0.59	0.46	0.11	0.10	88.2	0.87	45.0	3.48	2.05	1.20	–	0.052	0.004	13.3	50.3
W401	0.61	0.49	0.17	0.07	94.1	0.46	37.9	2.12	1.24	0.73	–	0.042	0.002	18.5	77.7
W64A	0.40	0.34	0.12	0.03	92.3	0.29	25.6	0.99	0.79	0.39	–	0.043	0.002	23.4	80.4
WF9	0.65	0.52	0.13	0.09	79.6	0.29	53.4	3.56	1.90	1.18	–	0.056	0.004	16.0	176.7
Mean	0.58	0.46	0.13	0.08	87.0	0.48	40.8	2.78	1.65	0.95	–	0.048	0.003	16.5	92.0
SD	0.11	0.08	0.02	0.03	7.2	0.18	12.8	1.1	0.55	0.35	–	0.014	0.001	6.7	43.6
CV%	19.2	17.7	17.0	34.0	8.2	38.4	31.3	40.9	33.2	37.1	–	29.1	32.2	40.3	47.3

*pCA, p-coumaric acid; FA_m_, ferulic acid monomer; diFA, ferulic acid dehydrodimers (8-5′, 8-O-4′, 5-5′); TriFA, ferulic acid dehydrotrimer; FA, total ferulic acid. The B73 line was used in the model study and is highlighted in the table*.

In each line, the pericarp mainly contained cell wall polysaccharides (average of approximately 70%) composed of arabinose, xylose and glucose, with a lower amount of galactose and trace amounts of mannose. The mean relative proportions of arabinose, xylose and galactose (expressed as weight %) are very close to the proportions observed for heteroxylans extracted and purified from maize bran (Chanliaud et al., 1995; Lapierre et al., 2001). Therefore, the arabinose, xylose and galactose contents can be mostly ascribed to heteroxylans, and it can be assumed that glucose mainly originates from cellulose and from a minor amount of mixed-linkage glucan. Surprisingly, correlation analysis of the cell wall composition of the 19 lines revealed that the arabinose and xylose contents were not highly correlated, and the galactose content was not linked to either the arabinose or xylose content (Supplementary Table 1). This might reflect variations in the extent of xylan substitution by single arabinose residues and complex side chains comprising arabinose, xylose and galactose residues. Regarding hydroxycinnamates, the ferulic acid monomer content estimated by the level of ferulic acid released upon alkaline hydrolysis was extremely high in the maize pericarp, ranging from 1.9 to 5.0%, with a mean value of 3.5%. Likewise, as previously reported (Saulnier and Thibault, 1999; Rouau et al., 2003; Bunzel et al., 2004), the amount of ferulic dimers and trimers was also high in maize pericarp. The relative proportions of the different dimers and trimers were stable among the maize lines. Their amounts were highly correlated with each other. However, ferulic dimers and trimers were not correlated with the content of ferulic acid monomer or with the content of other cell wall components. The amount of ferulic acid was not correlated with the arabinose content, and the molar ratio of ferulic acid to arabinose or of diferulate to arabinose showed a large variation among the different lines. On average, approximately 14% of the arabinose residues were ester-linked to ferulic acid, and approximately 2% of the arabinose residues were involved in the cross-linkage of heteroxylan chains through dimer and trimer bridges. The *p*-coumaric acid content was generally 10–20 times lower than that of ferulic acid. However, large variations in both *p*-coumaric and ferulic acid contents were observed between the different lines.

The pericarp is mainly composed of cell wall material, whereas the neutral sugar analysis of the maize endosperm showed a predominant level of starch-derived glucose (Table 2). Nevertheless, arabinose, xylose, galactose, and mannose were also detected, and the variation of their contents was highly correlated (Supplementary Table 1). The structure of maize endosperm xylan is unknown, but the ratio of arabinose to xylose (higher than 1) suggests a highly branched structure. This is in agreement with the structure reported for glucuronoarabinoxylans isolated from the endosperm of sorghum grains (Verbruggen et al., 1995). The arabinose and xylose contents are therefore probably mainly related to the same xylan polysaccharide, which may also contain glucuronic acid as reported for the endosperm of sorghum. These xylans contained a relatively high level of ferulic acid, but the molar ratio of ferulic acid to arabinose indicated that the extent of feruloylation was lower in endosperm xylans than in pericarp heteroxylans.

**Figure 2:**
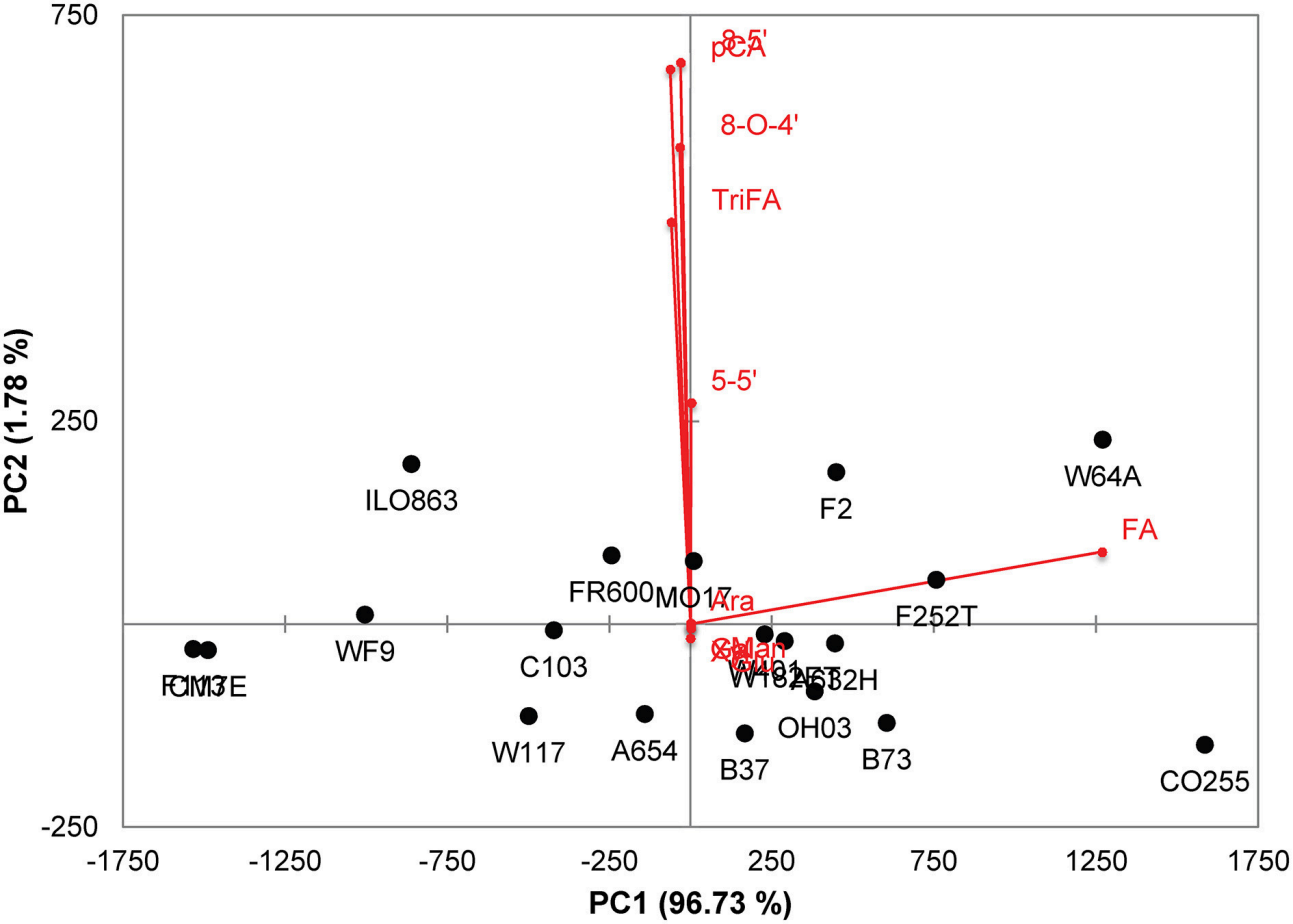
**Principal component analysis (PCA) of cell wall constituents (neutral sugar and hydroxycinnamic acids) in maize pericarps**. Bi-plot of loadings (red vectors and labels) and scores (black labels).

There was a considerable variability in the levels of the ferulic acid monomer and dimers in the grain tissues among maize lines as illustrated by the principal component analysis of the cell wall constituents of the pericarp (Figure 2). The two first principal components accounted for 98.6% of the total variance, and principal component 1 was mainly driven by the variation in the ferulic acid content (Figure 2). By contrast, component 2 was driven by variations in dimers that, as previously indicated, were not correlated with the ferulic acid content. The pericarp of several lines such as CO255, F252, W64A, B73, and F2 exhibited a very high amount of the ferulic acid monomer. However, the proportion of di/trimers of ferulic acid to the total amount of ferulic acid was low in these lines. The di-triFA/total FA was 19.1% for B73 and was 13% for CO255. This suggests that the extent of cross linking of arabinoxylan chains is not simply correlated with the monomeric ferulic acid content but may also depend on other factors such as the level of peroxidase activity in the cell wall and the distribution of ferulate monomers along the backbone, among others.

With respect to *p*-coumaric acid, the pericarp cell walls of B73 and CO255 contained notably low amounts of *p*-coumaric acid (0.62 and 0.51 mg.g^−1^, respectively) compared with the other lines (mean of 2.383 mg.g^−1^). The ratio of ferulic acid to *p*-coumaric acid (FA/*p*CA) was therefore high in the pericarp of B73 (67) and CO255 (94) compared with the mean ratio for the 19 lines (24). Interestingly, line W64A, which was also ferulic acid-rich, exhibited a high level of *p*-coumaric acid released from the pericarp (3.17 mg.g^−1^ and an FA/*p*CA ratio of 16).

Considerable variability exists in the ferulic and *p*-coumaric acid levels in the grain tissues of the maize lines. This variability is even more important when different tissues of other grass species are considered. Even in the same plant and organ (e.g., rice leaves from the same plant), the levels of ferulic and *p*-coumaric acids vary (Table 3). These contrasted values probably reflect functional differences or, at least in some cases, may be due to the difficulty in fully releasing hydroxycinnamates from lignified tissues.

**Table 3:** **Lignin and hydroxycinnamate contents of grass tissues (FT-IR reference samples and published reports)**.

**Sample**	**Lignin %**	**S/G ratio**	**FA mg/g**	***p*CA mg/g**	**FA_m_/*p*CA ratio**
Maize ear-bearing internode at silage stage F2 line (FT-IR reference sample) (1)	18.7 (KL)	0.9	5.3	18.3	0.29
Maize whole stem at silage stage F2 line (1)	16.4 (KL)	1	7.0	19.9	0.35
Wheat grain pericarp cultivar Baroudeur (2)	1–3 (thio; DM)	0.75	3.1 (DM)	na	na
Wheat grain pericarp cultivar Caphorn (3)	na	na	2.0 (DM)	0.05 (DM)	40.8
Wheat Caphorn grain starchy endosperm (3)	na	na	0.05 (DM)	0 (DM)	100
Wheat straw cultivar Champlein (FT-IR reference sample)	18.2 (KL)	1	2.7	4.8	0.56
Brachypodium mature stem Bd21-3 line (4)	19.6 (KL)	2.28	5.2	8.9	0.59
Brachypodium *pmt1* straw (FT-IR reference sample)	17.9% (KL)	2.0	5.0	1.9	2.63
Brachypodium root transition phase (5)	na	na	0.2	0.2	0.9
Rice stem (6)	na	na	6	5.5	1.09
Rice 3rd leaf 6 weeks old plants (6)	na	na	2	0.5	4
Rice 5th leaf 6 weeks old plants (6)	na	na	6	2	3
Rice leaf blades 7 weeks old plants (7)	na	na	2	1.5	1.33
Rice leaf blades 10 weeks old plants (7)	na	na	4	3	1.33
Rice leaf sheaths 7 weeks old plants (7)	na	na	1.5–3.5	1.7–3	0.9–1.2
Rice leaf sheaths 10 weeks old plants (7)	na	na	4–5.5	3.1–4	1.3–1.5
Maize destarched bran (8)	1–2 (thio)	4	31.0	4.0	7.75

*Analyses conducted on AIR samples except when stated DM (dry matter); Thio, thioacidolysis yield; KL, Klason lignin; na, not available. References: (1) Chazal et al., 2014; (2) Antoine et al., 2003; (3) Barron et al., 2007; (4) Ho-Yue-Kuang et al., 2016; (5) Molinari et al., 2013; (6) Piston et al., 2010; (7) Bartley et al., 2013; (8) Saulnier et al., 1995b, Lapierre et al., 2001*.

The B73 line was selected for further investigation due to its high ferulic acid content and high FA/*p*CA ratio and because it is the reference line for functional genomics in maize.

### FT-IR analysis confirms that maize pericarp has a low lignin content

Several studies have reported contrasting lignin contents for maize bran samples. Using the classical Klason method, we previously estimated the bran lignin content as 0.7–1.3% of the bran weight (Chanliaud et al., 1995). In a subsequent study using the thioacidolysis method that releases lignin monomers linked via β-*O*-4 linkages, this estimation was confirmed. Indeed, the yield of released monomers from destarched maize bran was found to be approximately one tenth of the yield obtained using maize internodes (16–17%) (Mechin et al., 2000; Lapierre et al., 2001).

A considerably higher amount of lignins (14%) was reported by Bunzel et al. (2004) in insoluble dietary fiber from maize bran. They cautiously stated that the acetyl bromide method they used led to an overestimation of the lignin content due to other compounds, such as hydroxycinnamates, which can be detected at the UV wavelength (280 nm) used to quantify the acetyl bromide lignin. Using the DFRC method (derivatization followed by reductive cleavage), which selectively cleaves β-*O*-4 linkages in lignins, the same authors obtained a low yield of lignin monomers. The low level of released monomers is in agreement with an overestimation of the acetyl bromide lignin content but could also be explained by a partial release of lignin monomers. A subsequent article reported that maize bran contains 10–14% lignin (Agger et al., 2010).

**Figure 3:**
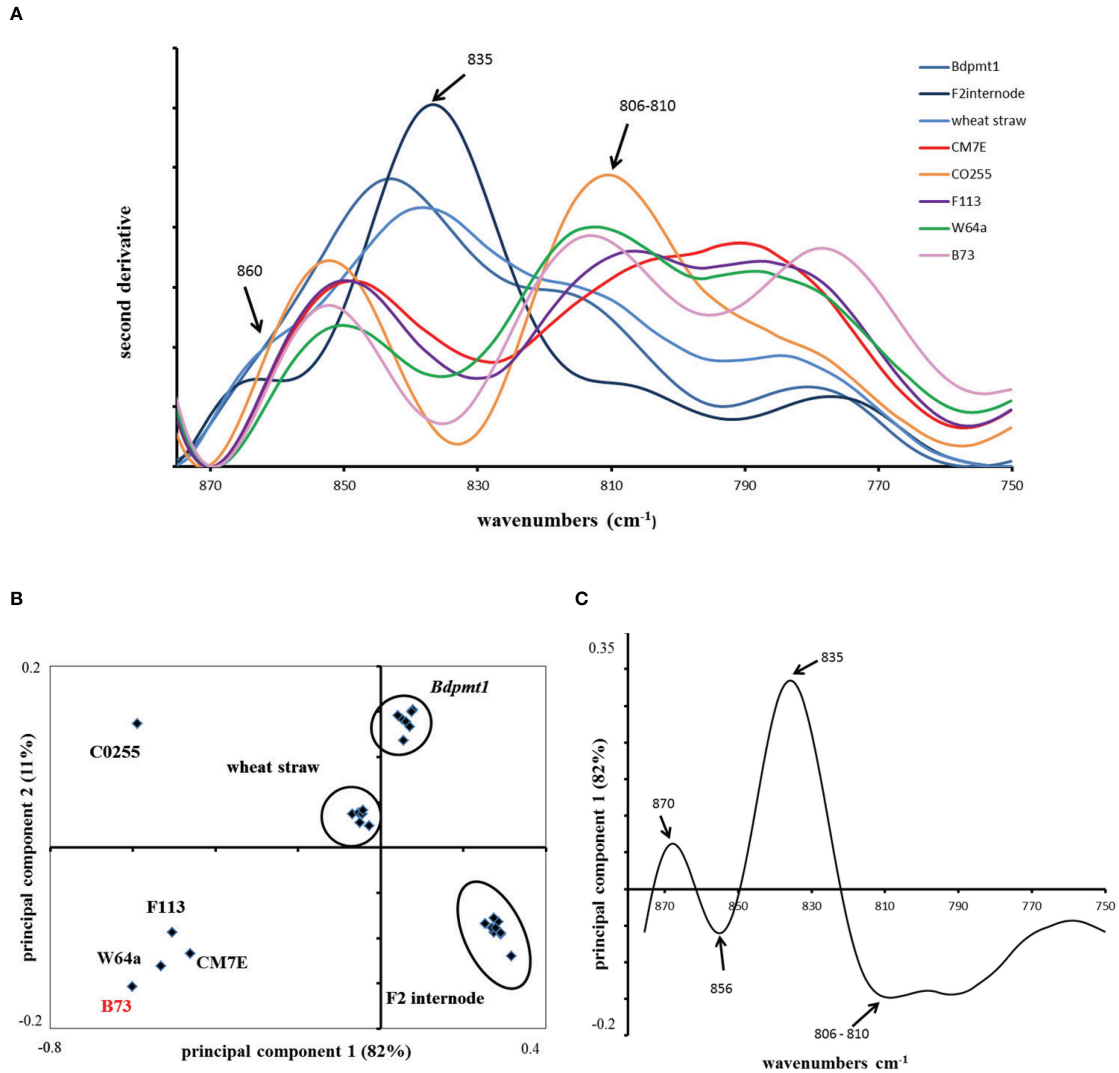
**Fourier Transform-InfraRed (FT-IR) analysis of maize pericarp. (A)** Second derivative of FT-IR spectra in the range of 875–750 cm^−1^ obtained for ground maize pericarp (B73, CM7E, CO255, F113, W64A lines) and reference samples rich in lignins: internode of maize F2 line, straw of wheat cv. Champlein, stem of Brachypodium mutant *Bdpmt1* (with low level of *p*-coumarate). The peak around 835 cm^−1^ is assigned to the S unit of lignin and to *p*-coumarate; the peak around 810 cm^−1^ is assigned to ferulate and the G unit of lignin. **(B)** Principal component analysis (PCA) of maize pericarp and reference samples. **(C)** Loading plot of principal component 1 showing that samples are distributed along PC1 according to ferulic acid and lignin contents.

Because of these contrasting values, we evaluated the lignin content of the pericarp of B73 and several other lines. We conducted Fourier transform infrared (FT-IR) analyses on ground maize pericarp. FT-IR has been shown to be a robust technique to study maize lignin and hydroxycinnamates (Chazal et al., 2014). The distinction between hydroxycinnamates and lignin in cell walls is complicated since no FT-IR peak can be exclusively assigned to *p*-coumarate, ferulate, or S or G monomers. However, using standards with a known lignin content and applying principal component analysis to second derivative spectra, it was possible to discriminate “lignin rich” and “lignin poor” samples by exploiting the 875–750 cm^−1^ region of the infrared spectra (Figure 3). Figure 3 shows the score plot defined by the first and second principal components. Principal component 1 explains 82% of the total variance and clearly separates the maize pericarp samples from the maize internode, the Brachypodium *pmt1* straw and the wheat straw, which all contained high amounts of lignins. More specifically, the band at 835 cm^−1^ was assigned to the S monomer and *p*-coumarate while the peaks at 810 and 860 cm^−1^ were assigned to the G monomer or ferulate (Sebastian et al., 2009; Chazal et al., 2014). Samples rich in lignin exhibited a band at 835 cm^−1^ for the maize F2 internode, wheat straw, and Brachypodium *pmt1* straw, while all of the maize pericarp samples exhibited a negative peak at approximately 835 cm^−1^, indicating a low amount of S monomer. CO255 and B73, which have a very low level of *p*-coumaric acid, were characterized by the most negative peaks. At 810 cm^−1^, the pericarp samples exhibited peaks that mirrored the amount of ferulic acid.

These data, and our previous results on maize bran, unambiguously indicate that the lignin content of maize pericarp is low, and this tissue provides an interesting model to decipher feruloylation of arabinoxylan in the cell walls of grasses.

### Feruloylated heteroxylans accumulate in pericarp cell walls during grain development (line B73)

The deposition of feruloylated heteroxylans in the cell walls of the B73 line was studied during grain development. Ears were harvested at the different stages of development described in Figure 1, and grains were collected to study the grain histology and to perform immunolabeling.

**Figure 4:**
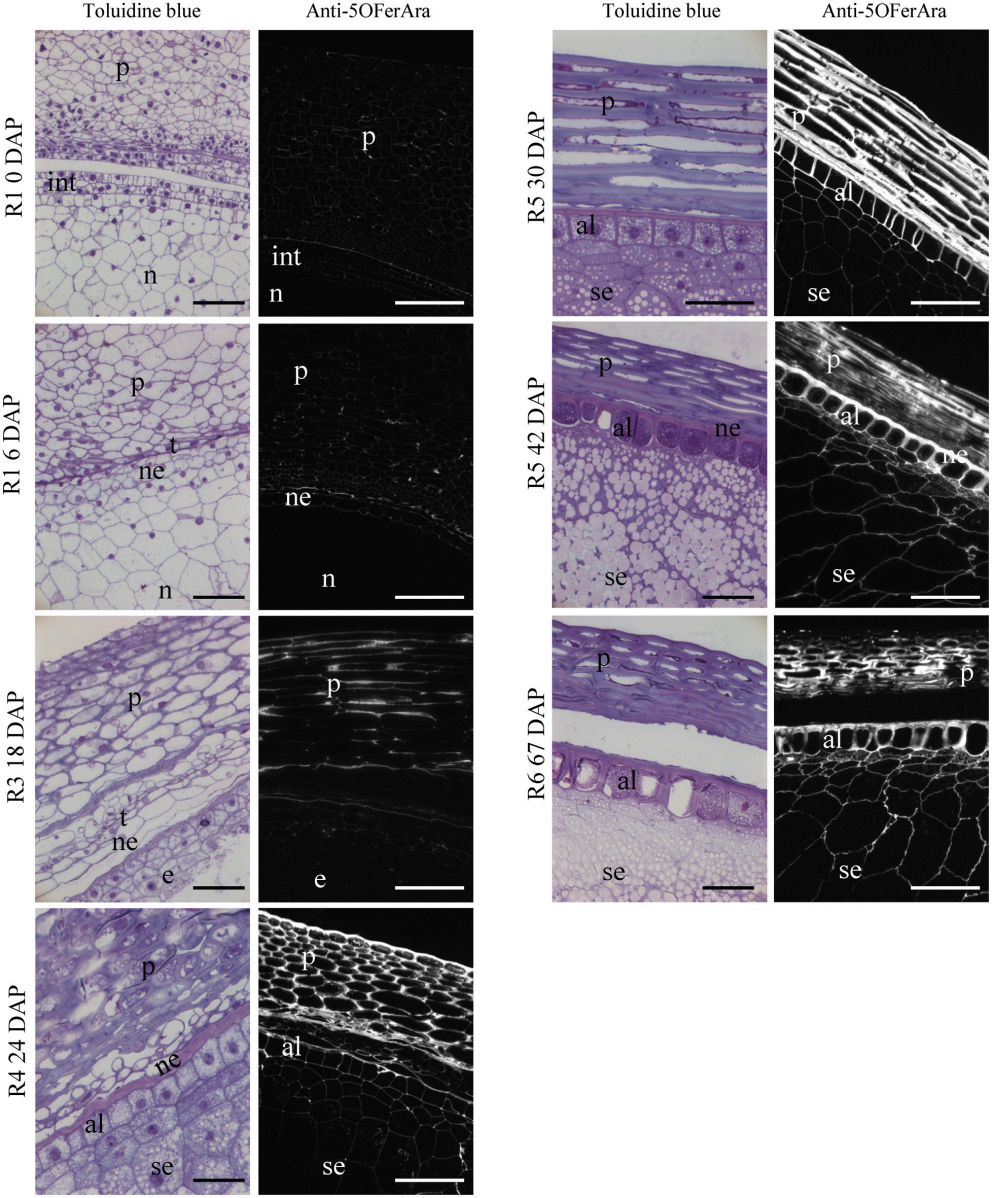
**Histology and detection of feruloylated arabinoxylans in the grain of maize B73 at different stages of development**. LR White-embedded longitudinal sections of maize grains stained with toluidine blue or labeled with anti-5-O-FerAra, an antibody targeting feruloylated arabinoxylans. Fluorescence images were captured using identical exposure conditions for all stages. p, pericarp; n, nucellus; e, endosperm; ne, nucellar epidermis; se, starchy endosperm; al, aleurone layer; t, testa; int, integuments. Bar = 50 μm.

Around fertilization at the beginning of stage R1, the observed grain tissues consisted of the pericarp, integuments and nucellus (Figure 4). Early in development, the integument layers evolve to form the testa, and the nucellus degenerates and is replaced by the endosperm; only the nucellus epidermis remains. The endosperm develops and then differentiates into the starchy endosperm and the aleurone layer clearly visible in the R4 stage (Figure 4). Late in development, the pericarp cell layers become compressed and starch fills the cells of the starchy endosperm.

The deposition of feruloylated heteroxylans in cell walls during grain development was investigated using the anti-5-O-FerAra antibody that specifically detects ferulate monomers ester-linked to arabinoxylans (Philippe et al., 2007). Negative control experiments were conducted using pre-immune sera instead of anti-5-O-FerAra, and no signal was observed (Supplementary Figure 1). A weak positive labeling was observed in the walls of the pericarp and the integuments around fertilization and during the pre-blister stage (Figure 4). The labeling strongly increases in the thickening walls of the pericarp from the R3 stage. At this stage the walls of the endosperm are not labeled (Figure 4). At the R5 stage, there was a strong positive signal in the thick cell walls of the pericarp, the walls of the aleurone layer and those of the starchy endosperm were also labeled, although the signal was weaker for the thin cell walls of the starchy endosperm. In the pericarp of nearly and fully mature grains (42 and 67 DAP), the signal intensity appeared weaker and more heterogeneous compared with observations in the previous developmental stages (Figure 4). A decrease in epitope accessibility could account for this reduced signal. This could result from a lower level of ferulate monomers due to oligomerization or other changes in the pericarp structure, such as tissue compression and drying.

These results suggest that feruloylated heteroxylans accumulate mainly in the pericarp cell walls during the R5 stage. At this stage, the maize grain has reached its final size, and ferulate mediated cross-linking of heteroxylans may stiffen the grain cell walls. Indeed, in another grass species (tall fescue), diferulate accumulation has been shown to correlate with growth cessation of leaves (MacAdam and Grabber, 2002).

### Gene expression analyses reveal that the phenylpropanoid pathway is directed to synthesize feruloylated arabinoxylans in maize grain pericarp

Taking advantage of the public microarray data available for maize B73, we analyzed the expression of gene families related to feruloylated heteroxylans and lignins. To identify candidate genes involved in heteroxylan synthesis and coupling of ferulic acid, we made a minimal number of assumptions. The maize grain pericarp is rich in feruloylated heteroxylans and poor in lignin; consequently, the metabolism in this tissue should be directed toward feruloylated heteroxylans. At the R3 stage when feruloylated heteroxylans have been detected in the pericarp but have not yet been detected in the endosperm (Figure 4), enzymes involved in their synthesis and coupling should be more represented in the pericarp than in the endosperm. Although no simple relationship exists between the amount of transcripts, the amount of enzymes and the amount of their product, the relative level of expression of the corresponding genes should, to some extent, reflect the relative amount of product.

The expression data for 60 maize tissues of the B73 line generated by Sekhon et al. (2011) and publicly available via the eFP Browser of the MaizeGDB website were used to study the expression of genes related to feruloylated heteroxylans and lignins. Data were selected for the dissected pericarp and endosperm at the R3 stage (18 DAP). The deposition of feruloylated heteroxylans occurs in the B73 pericarp during this stage. It is the only stage where both pericarp and endosperm data are available in the dataset from Sekhon et al. (2011). We also selected data for the 1st internode of the maize stem at stage V7 (7 leaves with visible leaf collars) since its cell walls contain glucuronoarabinoxylans and lignins (Scobbie et al., 1993; Guillaumie et al., 2007).

#### Heteroxylan-related genes

Several maize genes orthologous to Arabidopsis genes involved in the synthesis of the xylan backbone were found highly expressed in maize pericarp at the R3 stage (Supplementary Table 2 and short-list in Table 4). The xylan backbone is produced by a xylan synthase complex that has been shown to contain several proteins including glycosyltransferases of family 47 (GT47) and GT43 (Zeng et al., 2010, 2016; Jiang et al., 2016). In Arabidopsis, mutations in GT47 *IRREGULAR XYLEM10* (*IRX10*), GT43 *IRX9* and *IRX14* result in a reduced level of xylans (Brown et al., 2005, 2009). The respective functions of the GT47 and GT43 enzymes in xylan backbone synthesis are still under debate. IRX10 was shown to have xylosyltransferase activity while GT43/IRX9 was proposed to have a structural function in the complex (Ren et al., 2014; Urbanowicz et al., 2014; Zeng et al., 2016). At the R3 stage, several maize genes orthologous to AtIRX10, AtIRX9 and AtIRX14 were highly expressed in the maize pericarp (Table 4) and can be considered as good candidates to play a role in xylan synthesis. All of these genes were also highly expressed in maize internode 1 at stage V7 (Table 4), which contains xylans.

**Table 4:** **Short-list of genes potentially encoding enzymes involved in the biosynthesis and coupling of feruloylated heteroxylans and of monolignols in the maize pericarp, endosperm, and internode**.

	**Gene**	**% Expression potential**			**Linearized RMA signal**		
		**Pericarp**	**Endosperm**	**Internode 1 V7**	**Pericarp**	**Endosperm**	**Internode 1 V7**
**XYLAN**							
IRX10-like	GRMZM2G023020	80.4	52.9	69.7	2640	1737	2287
IRX10-like	GRMZM2G056702	96.9	44.4	84.9	15,812	7244	13,847
IRX10-like	GRMZM2G448834	42.4	10.9	52.7	1470	378	1826
IRX10-like	GRMZM2G059825	33.2	15.2	62.4	5367	2454	10,080
IRX14-like	GRMZM2G113655	84.5	63.2	85.8	51,791	38,754	52,639
IRX14-like	GRMZM2G150302	41.9	26.1	83.7	16,570	10,323	33,110
IRX9-like	GRMZM2G001079	62.8	6.4	46.8	17,970	1833	13,395
GUX1-like	GRMZM2G135743	87.8	29.5	50.0	21,521	7224	12,258
XAX1-like	GRMZM2G094579	81.8	28.4	22.8	605	210	168
XAX1-like	GRMZM2G098793	27.4	4.0	15.2	1918	283	1067
XAT1-like	GRMZM2G176576	90.0	29.1	30.7	13,151	4256	4481
Caps1 RWA1-4 like	GRMZM2G076394	39.1	0.5	0.4	6793	94	67
Caps1 RWA1-4 like	GRMZM2G020721	44.5	20.3	66.0	10,348	4720	15,372
**BAHD ACYL TRANSFERASE**							
BAHD clade A (III)	GRMZM2G108714	42.3	7.3	16.1	1934	332	738
BAHD clade A (III)	GRMZM2G060210	34.5	5.8	14.0	2956	494	1204
BAHD clade A (IV)	GRMZM2G314898	68.7	8.5	39.3	12,948	1598	7401
BAHD clade A (I)	GRMZM2G094428	31.3	7.1	62.9	1366	308	2747
BAHD clade A (I)	GRMZM2G050270	25.9	5.4	53.6	1766	365	3648
**PHENYLPROPANOID GENES**							
PAL3a (pal3 locus ZmPAL)	GRMZM2G074604	38.8	2.5	60.7	13,549	889	21,213
PAL3b (pal3 locus)	GRMZM2G029048	69.3	5.1	38.6	19,037	1408	10,595
PAL3c (pal3 locus)	GRMZM2G081582	86.6	5.0	42.6	9943	579	4887
PAL3d (pal3 locus)	GRMZM2G160541	28.9	2.5	49.3	9985	858	17,013
C3'H2	GRMZM2G140817	52.6	24.1	85.3	7137	3267	11,576
C4H3	GRMZM2G147245	23.3	4.3	23.8	7064	1307	7206
F5H1	AC210173.4_FG005	11.7	11.3	57.6	74	72	364
F5H2	GRMZM2G100158	3.8	2.6	71.2	102	69	1911
4CL1	GRMZM2G075333	30.7	1.5	55.8	6465	323	11,742
HCT2	GRMZM2G158083	63.6	21.3	35.1	3506	1175	1936
CCoAOMT1	GRMZM2G127948	82.2	22.4	78.3	45,732	12,444	43,555
CCoAOMT2	GRMZM2G099363	88.4	24.4	68.9	35,169	9702	27,402
CCoAOMT3	GRMZM2G004138	49.8	17.6	2.1	4531	1599	187
CCoAOMT5	GRMZM2G332522	17.3	0.9	44.1	2473	134	6311
ZmCCR1	GRMZM2G131205	9.4	1.9	42.1	1386	275	6221
ZmCCR2	GRMZM2G131836	12.3	5.9	54.5	67	32	296
ZmCCR3	GRMZM2G057328	3.8	3.2	18.1	157	132	744
ZmCCR4	GRMZM2G099420	2.1	0.2	0.3	257	29	39
COMT (bm3)	AC196475.3_FG004	35.6	3.6	77.0	15,863	1594	34,282
ZmCAD1	GRMZM2G179981	55.8	7.1	65.5	12,295	1571	14,437
CSE-like	GRMZM2G424577	20.3	14.8	12.7	688	500	429
ZmALDH RF2D	GRMZM2G097706	29.4	7.2	1.7	2686	658	155
ZmALDH RF2C	GRMZM2G071021	28.4	4.0	16.4	1408	200	811
**CLASS III PEROXIDASES**							
ZmPRX2	GRMZM2G047456	89.0	23.7	10.7	4582	1219	552
ZmPRX15	GRMZM2G341934	77.7	6.4	68.4	5877	485	5173
ZmPRX39	GRMZM2G321839	71.3	6.1	6.6	1750	150	162
ZmPRX66	GRMZM2G089895	81.4	4.2	9.7	3489	182	417
ZmPRX87	GRMZM5G843748	53.2	32.2	16.5	1584	958	491
ZmPRX117	GRMZM2G394500	41.1	1.7	18.1	2248	94	991

*% Expression potential with expression potential defined as the maximum absolute signal value in the maize RMA normalized data for 60 maize tissues of the B73 line (Sekhon et al., 2011, maize GDB), color-coded to facilitate visualization of expression differences. Linearized RMA signal: expression value (normalized data) selected for grain pericarp and endosperm at stage R3 and internode stage V7. In gray, value <200 not expressed*.

Regarding candidates for the different types of xylan substitutions, genes orthologous to wheat xylan O-3 arabinosyltransferase (XAT), rice arabinoxylan β-1,2-xylosyltransferase (XAX) and Arabidopsis xylan glucuronyltransferase (GUX) (Mortimer et al., 2010; Anders et al., 2012; Chiniquy et al., 2012) were also found highly expressed in maize pericarp (Table 4). However, no good candidate expressed in the pericarp was identified among maize orthologs of Arabidopsis glucuronic acid *O*-methyl transferase (GXMT) (Urbanowicz et al., 2012), which methylates glucuronic acid linked to xylans. Xylans from maize bran were indirectly shown to be acetylated (Agger et al., 2010). Three protein families have been demonstrated to play a role in xylan acetylation in Arabidopsis, and we investigated the expression of orthologs of these genes in the maize pericarp. REDUCED WALL ACETYLATION (RWA) proteins are proposed to be transporters of acetylCoA (Gille et al., 2011; Lee et al., 2011), ALTERED XYLOGLUCAN 9 (AXY9) protein is proposed to play a role in the supply of the acetyl donor (Schultink et al., 2015) and several DUF231 domain-containing proteins of the TRICHOME BIREFRINGENCE LIKE (TBL) family have been shown to be xylan acetyltransferases (Xiong et al., 2013; Urbanowicz et al., 2014; Yuan et al., 2016). In our analysis, we identified orthologs of RWA but no orthologs of AXY9 and TBL proteins were expressed in the maize pericarp. We speculate that either maize heteroxylans are not acetylated at the R3 stage or that the best homologs of Arabidopsis acetyltransferases are not involved in xylan acetylation in maize pericarp.

Nevertheless, several genes highlighted by our expression analysis encoded good candidates for heteroxylan-synthesizing enzymes in the maize pericarp.

#### General phenylpropanoid pathway (Figure 5)

**Figure 5:**
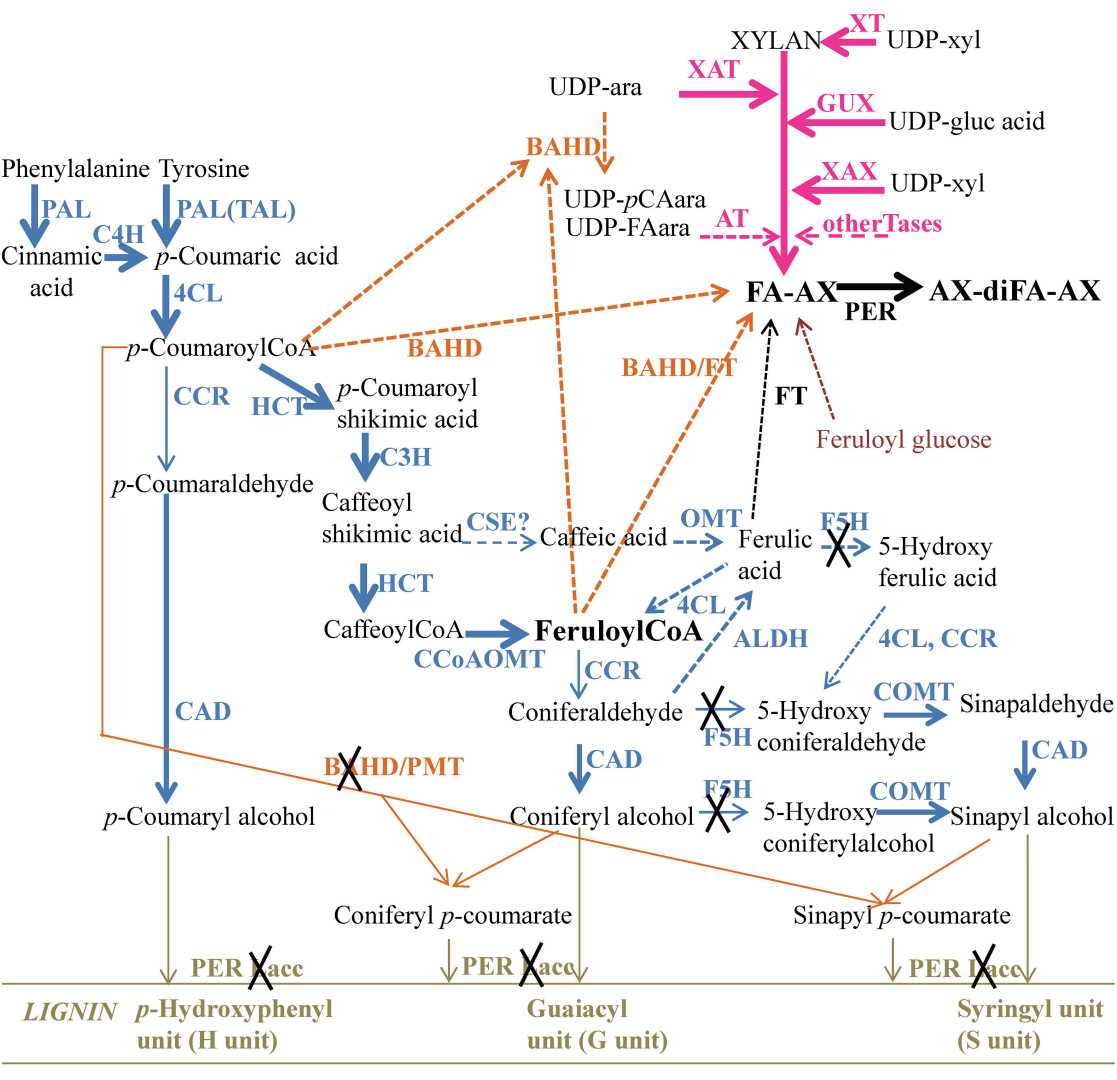
**Pathways leading to lignin and heteroxylan in maize pericarp at stage R3**. Enzymes and intermediate compounds are depicted. The thickness of the arrows represents the expression level of the genes known or proposed to carry out the activity in maize pericarp, while a cross means no expression. Dashed lines represent the proposed pathway but with no experimental evidence or unknown enzymes. PAL, phenylalanine ammonia lyase; TAL, tyrosine ammonia lyase; 4CL, 4-coumarate:CoA ligase; CCoAOMT, caffeoyl-CoA O-methyltransferase; COMT, caffeic acid O-methyltransferase; CCR, cinnamoyl-CoA reductase; CAD cinnamyl alcohol dehydrogenase; C4H, cinnamate 4-hydroxylase; C3H, p-coumarate 3-hydroxylase; HCT, hydroxycinnamoyl transferase; F5H, ferulate 5-hydroxylase; CSE, Caffeoyl shikimate esterase; ALDH, aldehyde dehydrogenase; PER: peroxidase; Lacc, laccase; XT, xylosyl transferase; XAT, xylan arabinosyltransferase; XAX, xylan xylosyltransferase; GUX, glucuronyltransferase; Tase, transferase; FT, feruloyltransferase; FA, ferulate; diFA, diferulate.

In the 1st internode of B73 at the V7 stage, many genes related to the phenylpropanoid pathway (Figure 5) leading to the synthesis of monolignols (lignin monomers) were expressed (Supplementary Table 2 and Table 4). Among them, there were several genes encoding phenylalanine ammonia lyases (PAL), three encoding a cinnamate 4-hydroxylase (C4H), one encoding a 4-coumarate:CoA ligase (4CL), two encoding a hydroxycinnamoyl transferase (HCT), two encoding a *p*-coumarate 3-hydroxylase (C3H), three encoding a caffeoyl-CoA *O*-methyltransferase (CCoAOMT), three encoding a cinnamoyl-CoA reductase (CCR), two encoding a ferulate 5-hydroxylase (F5H), one encoding a caffeic acid *O*-methyltransferase (COMT), and two encoding a cinnamyl alcohol dehydrogenase (CAD). Many of these genes were already revealed in a study on phenylpropanoid genes conducted on lignified internodes of the maize F2 line (Guillaumie et al., 2007), although the maize genome was not sequenced at that time.

Overall, the phenylpropanoid genes that were expressed in the 1st internode of B73 were also expressed in the pericarp at the R3 stage. Four PAL genes were highly expressed and more expressed than in the endosperm. Zm *C4H1, 4CL1, C3H2, HCT1* and *2*, and *CCoAOMT1* and *2* were well expressed in the pericarp and internode, and they were less expressed in the endosperm (Supplementary Table 2 and Table 4). Interestingly, two CCoAOMT genes (*CCoAOMT1* and *2*) were more highly expressed in the pericarp than in the internode, and their expression value for the pericarp is close to the maximum level observed in the 60 tissues investigated by Sekhon et al. (2011). By contrast, *CCoAOMT3* was well expressed in the pericarp but not expressed in the internode, and *CCoAOMT5* was more expressed in the internode than in the pericarp, showing rare examples of organ-specific expression among phenylpropanoid genes. The precise function of all of the individual CCoAOMT genes has not yet been elucidated. However, RNA interference (RNAi) lines targeting maize *CCoAOMT1* exhibited a 22% decrease in lignin content; therefore, at least CCOAOMT1 is involved in the synthesis of monolignols by producing feruloylCoA (Li et al., 2013). Together, these data suggest possible high production of feruloylCoA in maize pericarp (Figure 5).

Downstream of feruloylCoA, two gene families of the lignin pathway, F5H and CCR, exhibited low expression in the pericarp compared with the lignifying internode. CCR catalyzes the first step of monolignol-specific biosynthesis by transforming *p*-coumaroylCoA into *p*-coumaraldehyde, feruloylCoA into coniferaldehyde and 5-hydroxyferuloylCoA into 5-hydroxyconiferaldehyde. The CCR genes identified in maize were expressed at a low level in the pericarp compared with the internode: the expression value for *CCR1* was approximately 4.5-fold lower than that for the internode and 10-fold lower than the maximum value, and *CCR2* was not expressed in the pericarp. CCR has been shown to be a limiting enzyme for monolignol synthesis in eudicots (Piquemal et al., 1998; Goujon et al., 2003; Leplé et al., 2007). Tobacco plants down-regulated for CCR activity showed a reduced lignin level (Piquemal et al., 1998), and the cell walls of Arabidopsis *Atccr1*/*irx4* mutants contained 50% less lignin than those of wild type plants (Jones et al., 2001). The analysis of Arabidopsis antisense lines targeting *AtCCR1* suggested that the CCR substrate feruloyCoA was redirected to other ferulate-bound compounds (Goujon et al., 2003; Vanholme et al., 2012). It is noteworthy that in contrast to the observations cited above, the analysis of a maize insertion mutant targeting ZmCCR1 revealed only a slight (10%) global decrease in the lignin content while the *ZmCCR1* gene exhibited a 70% decreased expression level (Tamasloukht et al., 2011).

In addition to the CCR family, the other monolignol-related gene family down-regulated in maize pericarp is F5H. No *F5h* gene was found to be expressed in the maize pericarp at stage R3. As shown in Figure 4, F5H activity is required to synthesize the S monomer. Interestingly, in the Arabidopsis *ccr1* mutant, the *AtF5H1* gene was the only lignin-related gene that was considerably down-regulated (Vanholme et al., 2012).

In the maize pericarp at stage R3, the low expression of the CCR genes and the absence of expression of the F5H genes might limit the conversion of feruloylCoA to lignin monomers and then increase the pool of feruloylCoA to be transferred to heteroxylans.

#### FeruloylCoA or ferulic acid

Several hypotheses co-exist for the donor of the feruloyl moiety that is linked to arabinoxylans. In the current literature, the two most commonly cited donors are feruloylCoA and ferulic acid. As shown above, *CCoAOMT* genes are well expressed in the pericarp and therefore, feruloylCoA can be produced in this tissue. Ferulic acid might be produced by a coniferaldehyde dehydrogenase (ALDH) from coniferaldehyde as described in Arabidopsis (Figure 5; Nair et al., 2004). According to our expression survey, two genes homologous to Arabidopsis *ALDH REDUCED EPIDERMAL FLUORESCENCE1 (REF1)* (Nair et al., 2004) were expressed in the maize pericarp and were more highly expressed than in the endosperm and internode (Table 4). However, this step would require the conversion of feruloylCoA to coniferaldehyde by CCR, which is expressed at a relatively low level in the pericarp. Ferulic acid might result from the transformation of caffeic acid by the action of an O-methyl transferase (OMT), either a COMT or another OMT, possibly a ZRP4-type OMT (Barrière et al., 2016). We identified maize proteins sharing sequence similarity with AtCSE (caffeoyl shikimate esterase) (Vanholme et al., 2013) that converts caffeoyl shikimic acid into caffeic acid. However, the corresponding genes are expressed at a low level in maize pericarp. In addition, it has recently been reported that maize and Brachypodium only contain CSE-like genes, and protein extracts prepared from their stems exhibited very weak CSE activity (Ha et al., 2016). The maize *COMT/BROWN MIDRIB 3* gene is expressed in the grain pericarp; however, maize *bm3* mutants have decreased levels of the S unit of lignin, but there is no decrease in ferulic acid (Barrière et al., 2004). None of the ZRP4-type OMT genes investigated here were expressed in the pericarp. Therefore, the feruloylCoA hypothesis is strengthened by our maize pericarp expression survey.

#### BAHD genes

The BAHD acyltransferase family has been proposed to include AX feruloyltransferase that would transfer feruloylCoA to either arabinoxylans or UDP-arabinose (Mitchell et al., 2007; Molinari et al., 2013). Two clades of the BAHD family (named A and B) were identified in rice and Brachypodium as potentially encoding AX feruloyltransferases by the group of R. Mitchell (Mitchell et al., 2007; Molinari et al., 2013). Clade A was retained as a favorite candidate group because it contains genes that are co-expressed with genes related to arabinoxylan synthesis (Molinari et al., 2013). We collected the maize sequences of the BAHD family clade A. The phylogenetic tree provided in Supplementary Figure 2 shows the orthologous relationships between the sequences of maize, rice and Brachypodium BAHD proteins.

Five maize BAHD genes of clade A (GRMZM2G108714, GRMZM2G060210, GRMZM2G314898, GRMZM2G094428, GRMZM2G050270) were highly expressed in the pericarp at the R3 stage but less expressed in the endosperm. Consequently, these genes may be considered as good candidates for an AX feruloyltransferase function (Supplementary Table 2 and Table 4). These five genes were also well expressed in the 1st internode (Table 4). Therefore, the corresponding protein might also acylate maize stem glucuronoarabinoxylans with ferulic acid. Based only on the expression level, the best candidate would be GRMZM2G314898, i.e., the most highly expressed gene of BAHD clade A in the maize pericarp and internode. To further prioritize the candidate genes, their orthologous relationships with previously studied rice and Brachypodium genes were assessed. Clade A of Molinari et al. (2013) was divided into 4 subgroups (I, III, IV, V) in Piston et al. (2010) and Buanafina et al. (2016). Subgroup V includes rice and Brachypodium PMTs; therefore, not all clade A genes are involved in xylan acylation. None of the maize genes in subgroup V were expressed in the pericarp (Supplementary Table 2). Within subgroup I, GRMZM2G094428 and GRMZM2G050270 were highly expressed in the pericarp. Both genes were more expressed in the internode than in the pericarp. Nevertheless, the maize internode contained less ester-linked ferulic acid than the pericarp. This finding is not in favor of a function in xylan feruloylation. Piston et al. (2010) targeted rice genes of subgroup I (LOC_Os01g42870/*OsAT2* and LOC_Os01g42880/*OsAT1*) using a long silencing construct and did not observe any significant effect on the ester-linked ferulic acid level. However, the Brachypodium ortholog of LOC_Os01g42880 was targeted by Buanafina et al. (2016), who obtained RNAi lines and overexpressing lines, which, upon hydrolysis, showed respective decreased and increased release of monomers and dimers of ferulic acid from leaves and stems. Two maize genes of subgroup III, GRMZM2G108714 and GRMZM2G060210, were highly expressed in the pericarp and less expressed in the internode. Subgroups III and IV were targeted by Piston et al. (2010) using a long silencing construct. In the leaves of the RNAi lines, a 10–30% reduction in the level of ferulic acid was observed (no effect was noticed on the *p*-coumaric acid level). Subgroup IV contains the rice genes LOC_Os01g09010/*OsAT9* and LOC_Os06g39390/*OsAT10* studied by Bartley et al. (2013), who tentatively assigned an AX *p*-coumaroyltransferase function to OsAT10. Indeed, several lines overexpressing the *OsAT10* gene showed an important increase in *p*-coumaric acid ester-linked to cell walls in young leaves (a 300% increase in *p*-coumaric acid in one line) and a moderate decrease in ester-linked ferulic acid (Bartley et al., 2013). Interestingly, the maize ortholog of *OsAT10*, GRMZM2G107027, was not expressed in the pericarp of B73 (at the R3 stage). This is in agreement with the low level of *p*-coumaric acid found in the B73 pericarp (Table 1). By contrast, the maize ortholog of *OsAT9* is GRMZM2G314898, the maize BAHD gene of Mitchell's clade A with the highest expression value in the pericarp (Table 1). It therefore seems unlikely that GRMZM2G314898 also encodes an AX *p*-coumaroyltransferase. An AX feruloyltransferase function would be more likely since, in the pericarp of B73, the level of ferulic acid ester-linked to heteroxylans was considerably high compared with the level of *p*-coumaric acid (Table 1). In addition, according to Molinari et al. (2013), wheat orthologs to LOC_Os01g09010/*OsAT9* are expressed in wheat starchy endosperm where arabinoxylans contain ferulic acid but no *p*-coumaric acid. In the functional study of rice BAHD genes performed by Piston et al. (2010), LOC_Os01g09010/*OsAT9* and LOC_Os06g39390/*OsAT10* (and genes of subgroup III) were simultaneously down-regulated. A moderate reduction in the level of ferulic acid was measured while no effect on the *p*-coumaric acid level was observed. Therefore, several subgroups of clade A (I, III, IV) may contain AX feruloyltransferases. However, more experimental evidence is required to determine beyond any doubt which BAHD subgroup(s) has(have) such enzymatic activity since published RNAi experiments (Piston et al., 2010; Bartley et al., 2013) have not provided clear and consistent results.

**Figure 6:**
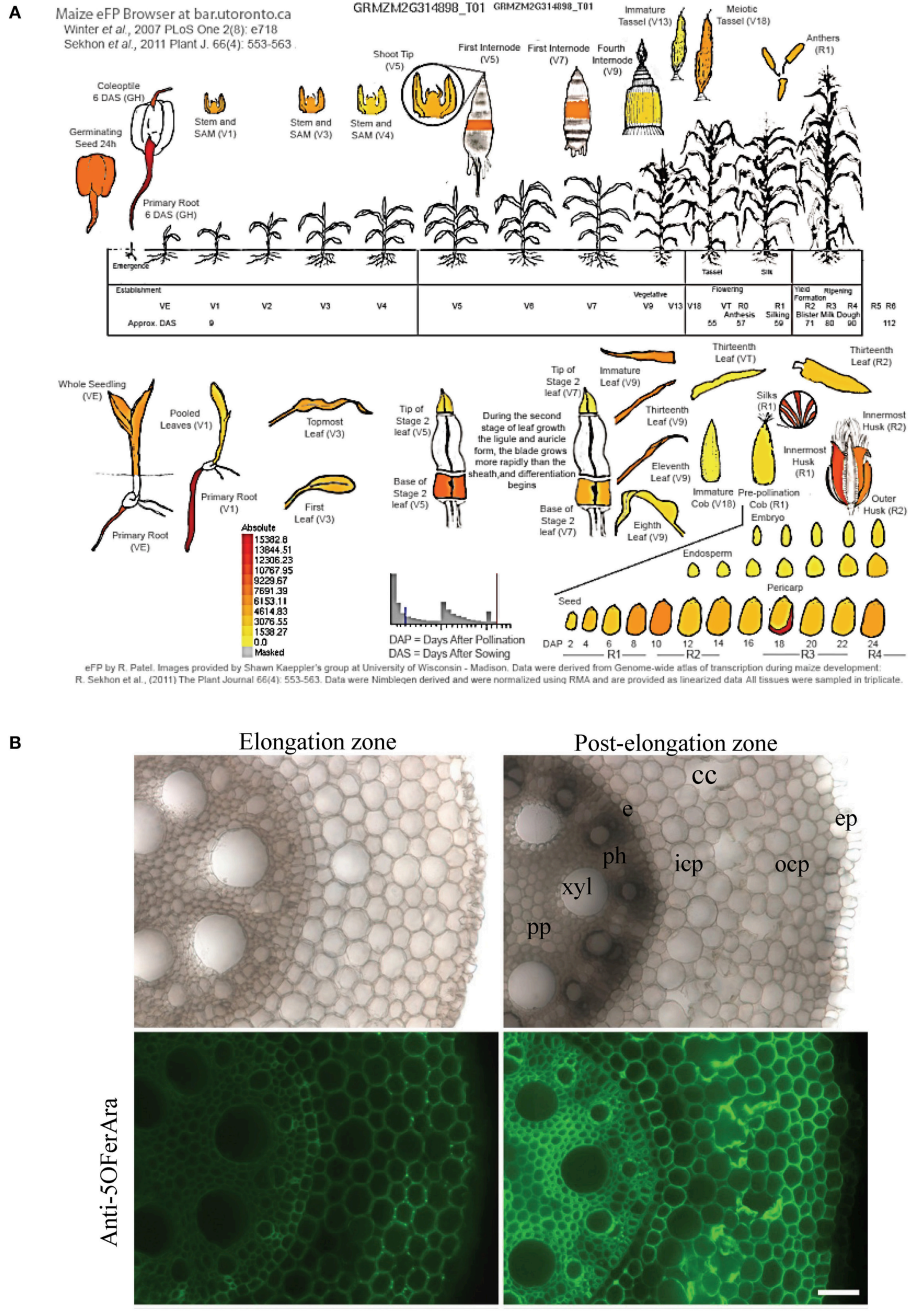
**The BAHD acyltransferase candidate heteroxylan feruloyltransferase is highly expressed in the maize pericarp and primary root where feruloylated heteroxylan is detected. (A)** Expression pattern of GRMZM2G314898 in B73 using the Maize eFP Browser (http://www.bar.utoronto.ca/). **(B)** Representative transverse sections of maize primary root (7-day-old plants) observed under brightfield illumination or labeled with an anti-5-O-FerAra polyclonal antibody targeting feruloylated arabinoxylans. To clearly visualize the immunodetection in the elongation zone, the intensity of fluorescence was increased in the micrograph. pp, pith parenchyma; xyl, xylem; e, endodermis; ph, phloem; icp, inner cortical parenchyma; ocp, outer cortical parenchyma; cc, cortical canal; ep, epidermis. Bar = 55 μm.

Considering the high expression level of GRMZM2G314898 in the pericarp and the considerably higher amount of ferulic acid compared with *p*-coumaric acid in the B73 pericarp, we select GRMZM2G314898 as the best candidate for involvement in heteroxylan feruloylation in maize pericarp. The highest expression of GRMZM2G314898 was measured in maize primary root. Glucuronoarabinoxylans have been detected in maize primary root (Kozlova et al., 2014), and maize orthologs to *IRX14* (GRMZM2G113655) and *GUX1* (GRMZM2G135743) are also highly expressed in maize primary root together with a putative *XAT* (GRMZM2G096946) (Supplementary Figure 3). To confirm that glucuronoarabinoxylans are feruloylated in maize root, we conducted immunolabeling with the anti-5O-FerAra polyclonal antibody on two distinct developmental regions of the maize primary root (elongation and post-elongation zone) (Figure 6). Although the intensity of detection was stronger in the mature region compared with the youngest region, the feruloylated arabinoxylan epitope was detected in almost every cell type in both zones. No signal was detected in control experiments (Supplementary Figure 1). These results support a role for GRMZM2G314898 in xylan feruloylation and its orthologs in other grass species.

#### Peroxidases

Peroxidases and laccases have been implicated in ferulic acid oxidative coupling and in monolignol coupling and lignin polymerization (for review, see Wang et al., 2013). To our knowledge, no peroxidase or laccase gene has been shown to be specifically involved in ferulic acid coupling. As the pericarp has a low lignin content but is rich in ferulic acid and dimers and trimers of ferulic acid resulting from its oxidative coupling, we examined the relative expression levels of genes encoding putative peroxidases and laccases. The genes encoding putative laccases investigated here were weakly expressed in the pericarp. Wang et al. (2015) identified 119 class III peroxidase genes in the maize genome. At least another locus encodes a class III peroxidase that was purified from the maize kernel and for which the peroxidase activity was demonstrated (López-Castillo et al., 2015). Among these 120 genes, 29 were expressed in the pericarp, and nine of them displayed a higher expression level in the pericarp than in the endosperm (GRMZM2G047456, GRMZM2G341934, GRMZM2G108153, GRMZM2G321839, GRMZM2G085967, GRMZM2G150893, GRMZM2G089895, GRMZM5G843748, GRMZM2G394500). Interestingly, co-expression analysis using the PlexdB platform revealed that GRMZM2G047456 is co-expressed with XAT/GRMZM2G176576, suggesting a possible role related to xylan.

## Conclusion

The pericarp of maize grain contains a high amount of heteroxylan highly substituted by arabinose and ferulic acid, as well as a low amount of lignin. Consequently, this tissue is a good model to study the biosynthesis, feruloylation and cross-linking of arabinoxylans. Here, we outlined how hydroxycinnamates that are ester-linked to cell walls can vary between plant species, genotypes and tissues. Our study reveals that the amount of ferulate dimers and trimers that reflects polymer crosslinking is not simply correlated with the level of ferulate monomers, which emphasizes a complex regulation of the feruloylation and coupling of arabinoxylans. To help elucidate the mechanisms controlling these traits, we propose candidate genes that potentially encode enzymes for heteroxylan synthesis, feruloylation and coupling in maize pericarp based on biochemical, immunolabeling and gene expression analyses. To clearly demonstrate the functions of these candidate genes, additional *in vivo* and *in vitro* studies are required. The effects of mutations and deletions of these genes could be investigated with the promising CRISPR/Cas9 technology and the substrate and acceptor preferences of the candidate proteins investigated using recombinant proteins. Feruloylation of grass cell walls impacts agriculture (pathogen resistance, silage digestibility), industries (bread making quality, biomass conversion), and human health (dietary fiber). The genes highlighted in this study as well as their close homologs may be interesting targets for grass breeding.

## Author contributions

ALCB and LS designed the research, CA, BB, JO, SD, YV, and ALCB conducted the experiments, JO, LS, ALCB, SD, and YB analyzed the data, ALCB, LS, YV, YB, CA, BB, SD wrote the manuscript. All authors read and approved the final manuscript.

### Conflict of interest statement

The authors declare that the research was conducted in the absence of any commercial or financial relationships that could be construed as a potential conflict of interest.

## Abbreviations

MaizeGDB: maize genetics and genomics database
DAP: days after pollination
FAm: ferulic acid monomer
diFA: ferulic acid dehydrodimers
TriFA: ferulic acid dehydrotrimer
AIR: alcohol insoluble residue
PMT: *p*CA monolignol transferase
FT-IR: Fourier transform-InfraRed
RMA: robust multiarray average
*p*CA: *p*-coumaric acid
GT: glycosyltransferase
IRX: IRREGULAR XYLEM
XAT: xylan O-3 arabinosyltransferase
XAX, arabinoxylan β-1: 2-xylosyltransferase
GUX: xylan glucuronyltransferase
GXMT: glucuronic acid O-methyl transferase
RWA: REDUCED WALL ACETYLATION
AXY: ALTERED XYLOGLUCAN
DUF: domain of unknown function
TBL: TRICHOME BIREFRINGENCE LIKE
PAL: phenylalanine ammonia lyase
C4H: cinnamate 4-hydroxylase
4CL: 4-coumarate:CoA ligase
HCT: hydroxycinnamoyl transferase
C3H: *p*-coumarate 3-hydroxylase
OMT, O-methyl transferase, CCoAOMT: caffeoyl-CoA *O*-methyltransferase
CCR: cinnamoyl-CoA reductase
F5H: ferulate 5-hydroxylase
COMT: caffeic acid *O*-methyltransferase
CAD: cinnamyl alcohol dehydrogenase
RNAi: RNA interference
ALDH: coniferaldehyde dehydrogenase
AX: arabinoxylan
CSE: caffeoyl shikimate esterase.
