# Supplementary material for: Developing Pericarp of Maize: A Model to Study Arabinoxylan Synthesis and Feruloylation
Source: Front Plant Sci. 2016 Sep 30;7:1476. doi: 10.3389/fpls.2016.01476 (PMC5043055; doi:10.3389/fpls.2016.01476)
Supplement: Supplementary file 3 [file Presentation1.PPTX]

## Slide 1
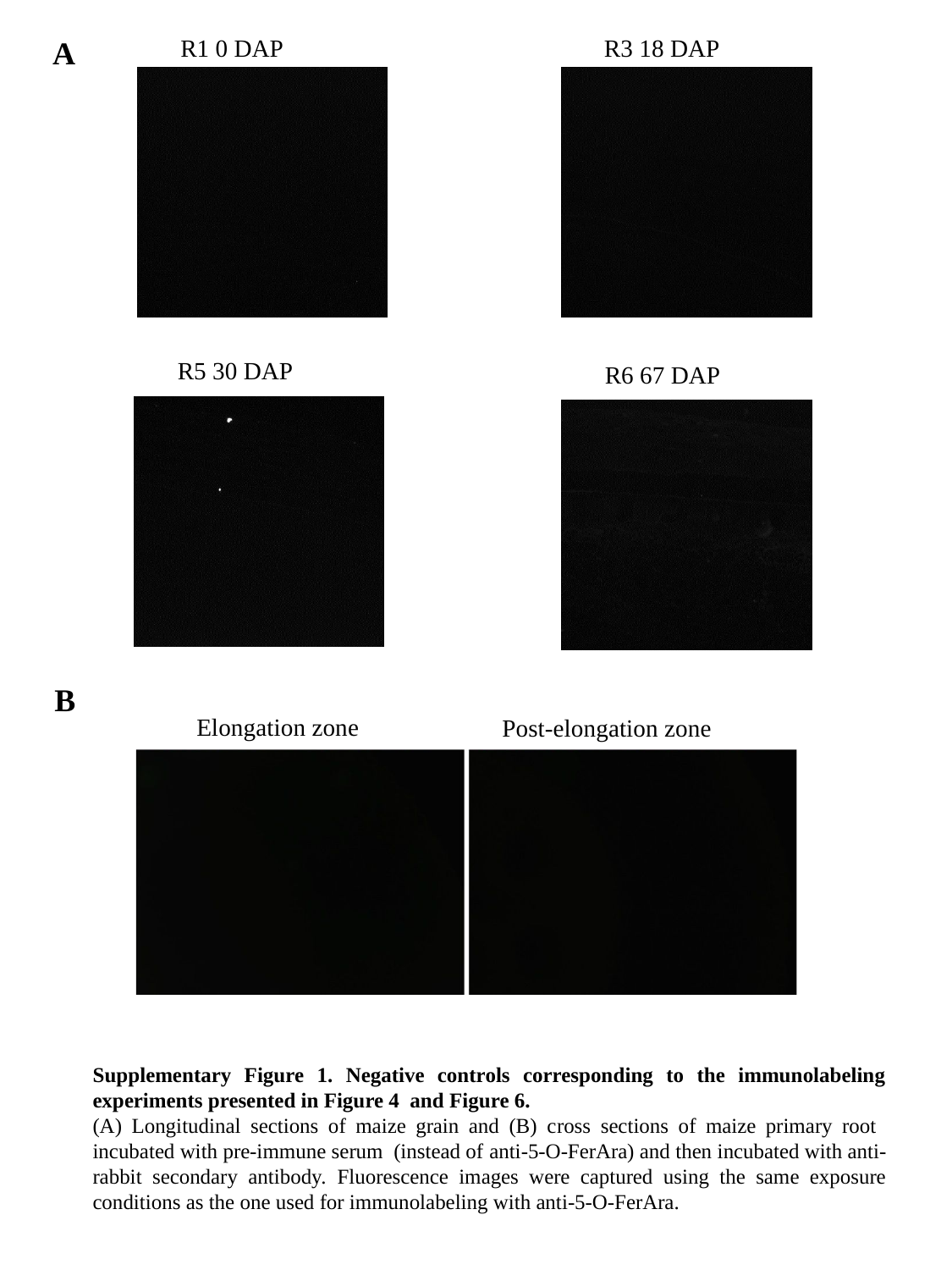

R1 0 DAP
R3 18 DAP
A
R5 30 DAP
R6 67 DAP
B
Elongation zone
Post-elongation zone
Supplementary Figure 1. Negative controls corresponding to the immunolabeling experiments presented in Figure 4 and Figure 6.
(A) Longitudinal sections of maize grain and (B) cross sections of maize primary root incubated with pre-immune serum (instead of anti-5-O-FerAra) and then incubated with anti-rabbit secondary antibody. Fluorescence images were captured using the same exposure conditions as the one used for immunolabeling with anti-5-O-FerAra.
