# Supplementary material for: Developing Pericarp of Maize: A Model to Study Arabinoxylan Synthesis and Feruloylation
Source: Front Plant Sci. 2016 Sep 30;7:1476. doi: 10.3389/fpls.2016.01476 (PMC5043055; doi:10.3389/fpls.2016.01476)
Supplement: Supplementary file 4 [file Presentation2.PPTX]

## Slide 1
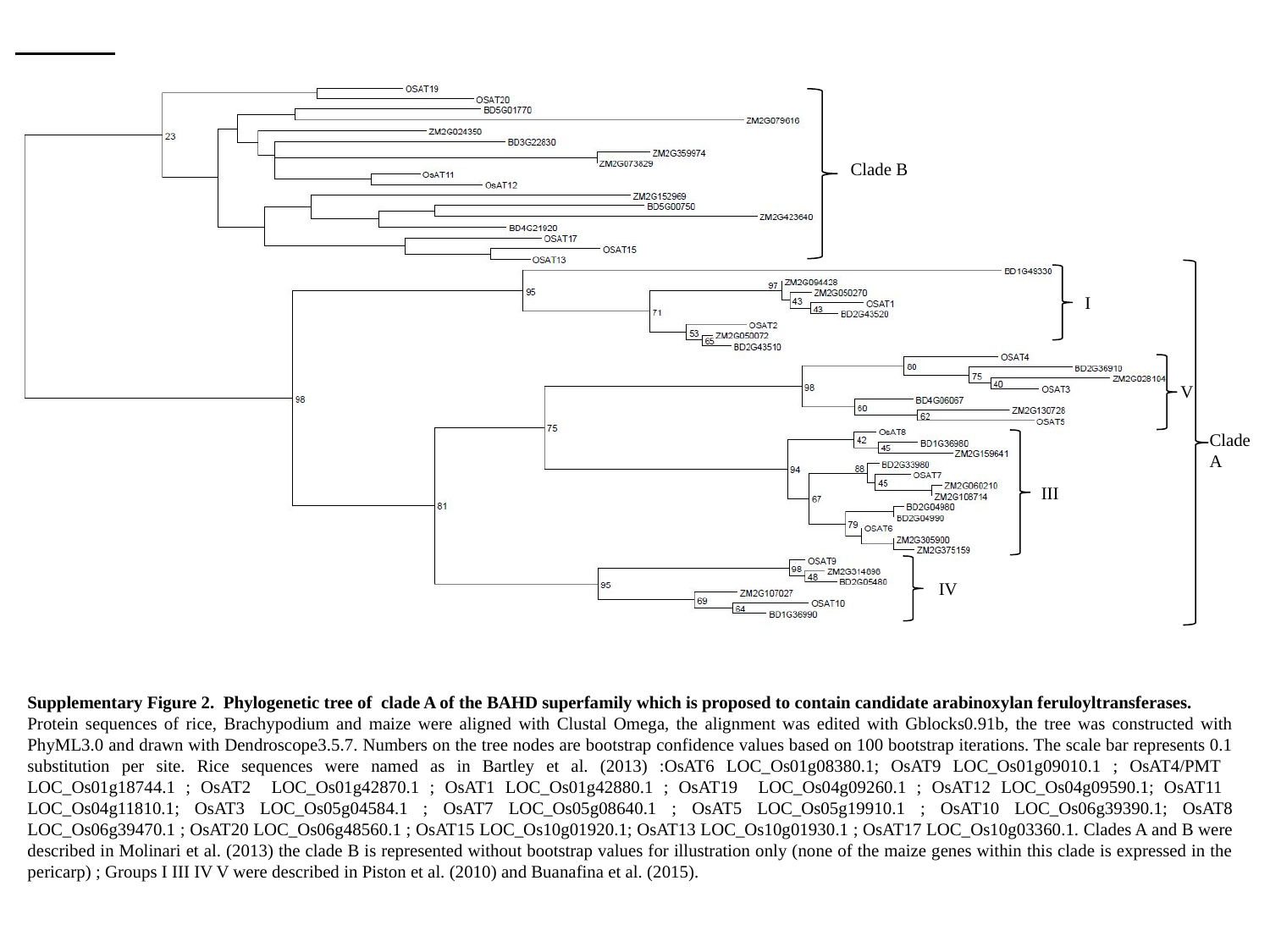

Clade B
I
V
Clade
A
III
IV
Supplementary Figure 2. Phylogenetic tree of clade A of the BAHD superfamily which is proposed to contain candidate arabinoxylan feruloyltransferases.
Protein sequences of rice, Brachypodium and maize were aligned with Clustal Omega, the alignment was edited with Gblocks0.91b, the tree was constructed with PhyML3.0 and drawn with Dendroscope3.5.7. Numbers on the tree nodes are bootstrap confidence values based on 100 bootstrap iterations. The scale bar represents 0.1 substitution per site. Rice sequences were named as in Bartley et al. (2013) :OsAT6 LOC_Os01g08380.1; OsAT9 LOC_Os01g09010.1 ; OsAT4/PMT LOC_Os01g18744.1 ; OsAT2 LOC_Os01g42870.1 ; OsAT1 LOC_Os01g42880.1 ; OsAT19 LOC_Os04g09260.1 ; OsAT12 LOC_Os04g09590.1; OsAT11 LOC_Os04g11810.1; OsAT3 LOC_Os05g04584.1 ; OsAT7 LOC_Os05g08640.1 ; OsAT5 LOC_Os05g19910.1 ; OsAT10 LOC_Os06g39390.1; OsAT8 LOC_Os06g39470.1 ; OsAT20 LOC_Os06g48560.1 ; OsAT15 LOC_Os10g01920.1; OsAT13 LOC_Os10g01930.1 ; OsAT17 LOC_Os10g03360.1. Clades A and B were described in Molinari et al. (2013) the clade B is represented without bootstrap values for illustration only (none of the maize genes within this clade is expressed in the pericarp) ; Groups I III IV V were described in Piston et al. (2010) and Buanafina et al. (2015).
