# Supplementary material for: Developing Pericarp of Maize: A Model to Study Arabinoxylan Synthesis and Feruloylation
Source: Front Plant Sci. 2016 Sep 30;7:1476. doi: 10.3389/fpls.2016.01476 (PMC5043055; doi:10.3389/fpls.2016.01476)
Supplement: Supplementary file 5 [file Presentation3.PPTX]

## Slide 1
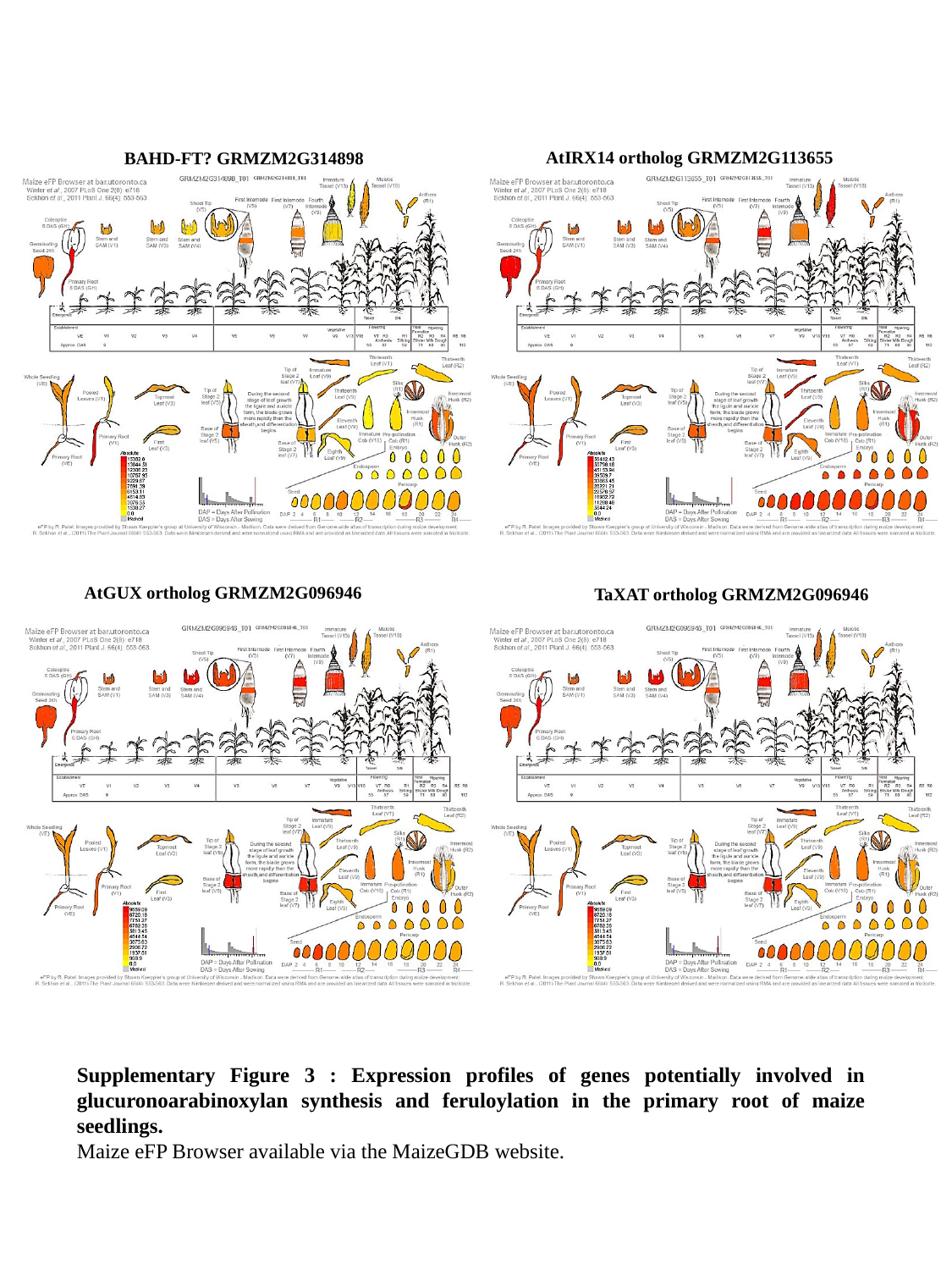

AtIRX14 ortholog GRMZM2G113655
BAHD-FT? GRMZM2G314898
AtGUX ortholog GRMZM2G096946
TaXAT ortholog GRMZM2G096946
Supplementary Figure 3 : Expression profiles of genes potentially involved in glucuronoarabinoxylan synthesis and feruloylation in the primary root of maize seedlings.
Maize eFP Browser available via the MaizeGDB website.
